# Isolation of nucleic acids using liquid–liquid phase separation of pH-sensitive elastin-like polypeptides

**DOI:** 10.1038/s41598-024-60648-9

**Published:** 2024-05-02

**Authors:** Telmo Díez Pérez, Ashley N. Tafoya, David S. Peabody, Matthew R. Lakin, Ivy Hurwitz, Nick J. Carroll, Gabriel P. López

**Affiliations:** 1 Center for Biomedical Engineering, University of New Mexico, Albuquerque, NM 87131 USA; 2 Center for Micro-Engineered Materials, University of New Mexico, Albuquerque, NM 87131 USA; 3 Department of Chemical and Biological Engineering, University of New Mexico, Albuquerque, NM 87131 USA; 4 Department of Molecular Genetics and Microbiology, University of New Mexico, Albuquerque, NM 87131 USA; 5 Department of Computer Science, University of New Mexico, Albuquerque, NM 87131 USA; 6 Department of Internal Medicine, Center for Global Health, University of New Mexico Health Sciences Center, Albuquerque, NM 87131 USA

**Keywords:** Characterization and analytical techniques, Biochemical assays

## Abstract

Extraction of nucleic acids (NAs) is critical for many methods in molecular biology and bioanalytical chemistry. NA extraction has been extensively studied and optimized for a wide range of applications and its importance to society has significantly increased. The COVID-19 pandemic highlighted the importance of early and efficient NA testing, for which NA extraction is a critical analytical step prior to the detection by methods like polymerase chain reaction. This study explores simple, new approaches to extraction using engineered smart nanomaterials, namely NA-binding, intrinsically disordered proteins (IDPs), that undergo triggered liquid–liquid phase separation (LLPS). Two types of NA-binding IDPs are studied, both based on genetically engineered elastin-like polypeptides (ELPs), model IDPs that exhibit a lower critical solution temperature in water and can be designed to exhibit LLPS at desired temperatures in a variety of biological solutions. We show that ELP fusion proteins with natural NA-binding domains can be used to extract DNA and RNA from physiologically relevant solutions. We further show that LLPS of pH responsive ELPs that incorporate histidine in their sequences can be used for both binding, extraction and release of NAs from biological solutions, and can be used to detect SARS-CoV-2 RNA in samples from COVID-positive patients.

## Introduction

The separation of nucleic acids (NAs), such as DNA and RNA, from biological samples, known as NA extraction or isolation, is a necessary first step in many analytical, diagnostic, molecular biological, and forensic procedures^1,2^. The NA isolation process typically involves several critical steps, including inactivation of resident nucleases to preserve NA integrity, cellular disruption, separation of the NA from cellular contaminants, and concentration of the extracted NA for further analysis^3^. Since the first DNA extraction in 1869, there has been significant progress in designing more affordable, efficient, and reliable methods^4^. Current commonly used processes can be categorized into two general types. A common example of liquid–liquid extraction is guanidium thiocyanate-phenol–chloroform extraction^1,5^. Solid-phase extraction methods use silica-based, microchromatographic columns (e.g., “spin columns”) or charged magnetic beads^6,7^. The stringency of washes used in these commercially available kits allows the isolation of the required NA. However, these systems continue to be problematic for some biologically relevant liquid samples such as sputum, blood and urine^8,9^. Although NA extraction continues to evolve and improve into more efficient and user-friendly processes, no universally established standardized technique is used in every application context. Available techniques vary in processing time, instrumentation, use of hazardous reagents, trained personnel, and laboratory infrastructure, each potentially impeding implementation in low-resource settings and miniaturized point-of-care devices^10–12^. Further, over-reliance on a few, specific extraction methods caused significant supply chain issues during the COVID pandemic, and so there is a significant incentive to diversify the range of options available to limit the impact of such issues in the future^13,14^.

This study investigates the use of engineered smart nanomaterials that can undergo triggered aqueous liquid–liquid phase separation (LLPS) for the isolation of NAs in a number of different solution contexts of biomedical relevance. We genetically engineered NA-binding, intrinsically disordered proteins (IDPs) based on elastin-like-polypeptides (ELPs, comprising V-P-G-X_1_-G amino acid repeats, where X_1_ is a guest residue) that undergo reversible phase separation in water characterized by a lower critical solubility temperature (LCST)^15^. We selected ELPs as model IDPs to engineer proteins that bind NAs to form nucleic acid-rich coacervates due to their modularity, versatility, and predictable phase behavior^16–19^. The LCST behavior of ELPs has been studied extensively via experiment and simulation as reviewed recently^20–22^. Condensation of solvated ELPs in water above a characteristic transition temperature (for a given polymer volume fraction) is thought to occur via increased ELP hydrophobicity at elevated temperatures (i.e., a temperature dependent reduction in solvent quality) and enhanced by favorable entropic contributions associated with release of bound water molecules upon polymer self-association. The hydropathy and associated temperature-controlled LLPS behavior of ELPs can be modulated by selection of the guest residue X and ELP molecular weight. Moreover, ELPs can be genetically engineered as fusions with other peptides, which can tailor the NA-binding function of these polymers while maintaining the temperature-triggered phase separation behavior of ELPs^16–18^. Liquid–liquid phase separation of NA-binding proteins is an attractive alternative for NA extraction since it does not require expensive equipment, hazardous reagents, or extensive technical expertise. By designing and expressing thermally responsive ELP-containing proteins that also bind NAs, our hypothesis is that a simple temperature increase can be used to trigger ELP LLPS and rapidly extract bound DNA or RNA from solution though gravitational settling of the dense nucleoprotein coacervate phase within the sample. Our previous work demonstrated the general strategy of this approach with an electrostatically charged, weak NA-binding ELP, which we call E3, for straightforward capture of oligomeric DNA in complex coacervates and its subsequent release in buffer solutions containing a screening electrolyte^23^. Here, we study a range of more robust NA-binding ELPs and their NA-binding and release activities in pseudo-clinical and clinical samples, as well as their compatibility with downstream RT-qPCR detection.

We used two approaches to engineer NA-binding ELPs and examined their efficiency in recruiting DNA and RNA species into protein-rich complex coacervates upon temperature-induced phase separation. First, we designed and studied the NA complexation behavior of two fusion proteins of ELP sequences and natural NA-binding motifs which we denote as E3.10 and E1-40.COR30 (Table 1). The E3.10 fusion comprises the previously studied, weak DNA-binding ELP, designated as E3 (sequence: [(VPGX_1_G)_10_-GKG]_8_, where X_1_ represents an 8:2 ratio of V:A)^24^, and RRM and RGG domains from the FUS protein attached at the C-terminus of the E3 sequence. The E1.40COR30 fusion comprises a 30 amino acid NA binding domain derived from the hepatitis C virus (HCV) core protein (HCV CP), fused at the C-terminus of the canonical ELP sequence E1.40 (i.e., (VPGVG)_40_). These two different ELP fusions exhibit NA capture upon temperature-induced coacervation. However, the triggered dissociation of these ELPs from NAs turned out to be challenging and dependent on the use of denaturants or detergents which can interfere with downstream processing (e.g., RT-PCR).

**Table 1:** Amino acid sequences of the elastin-like polypeptides and fusion proteins used in this study.

Protein	Amino acid sequence	X_1_	Length (aa)
E3	[(VPGX_1_G)_10_-GKG]_8_	V (80%), A (20%)	426
E3.10	[(VPGX_1_G)_10_-GKG]_8_-NTIFVQGLGENVTIESVADYFKQIGIIKTNKKTGQPMINLYTDRETGKLKGEATVSFDDPPSAKAAIDWFDGKEFSGNPIKVSFATR (*aka* RRM)- RADFNRGGGNGRGGRGRGGPMGRGGYGGGGSGGGGRGGFPSGGGGGGGQQR (*aka* RGG)	V (80%), A (20%)	564
E1.40COR30	(VPGVG)_40_-STNPKPQRKTKRNTNRRPQDVKFPGGGQIV (*aka* COR30)	V	231
H-20	MGH-[GVGVP GHGVP GGGVP GHGVP GAGVP]_20_-GW	V (20%), H (40%), G (20%), A (20%)	505
H-24	MGH-[GVGVP GHGVP GGGVP GHGVP GAGVP]_24_-GW	V (20%), H (40%), G (20%), A (20%)	605

To overcome this limitation, we synthesized NA-binding ELPs that exhibit pH-controllable electrostatic interactions. We hypothesized that ELPs that include multiple histidines (His) as guest residues (His-ELPs) would be promising candidates for controllable binding, coacervation and release of NAs in biological solutions because the pH-dependent protonation and associated charge state of His residues (i.e., cationic versus neutral)^25–27^ can impart pH responsiveness to the protein polymer to control ELP-NA electrostatic binding affinity and LLPS behavior. We synthesized two previously studied His-ELPs with the same guest residue composition (i.e., X = V (20%), H (40%), G (20%), A (20%)), but different chain lengths to study the electrostatic binding of the protein polymers with NAs as a function of pH^28^. The two His-ELPs comprise 20 or 24 repeats of 5 GXGVP pentamers and are referred to here as H-20 and H-24, respectively (Table 1).

**Figure 1:**
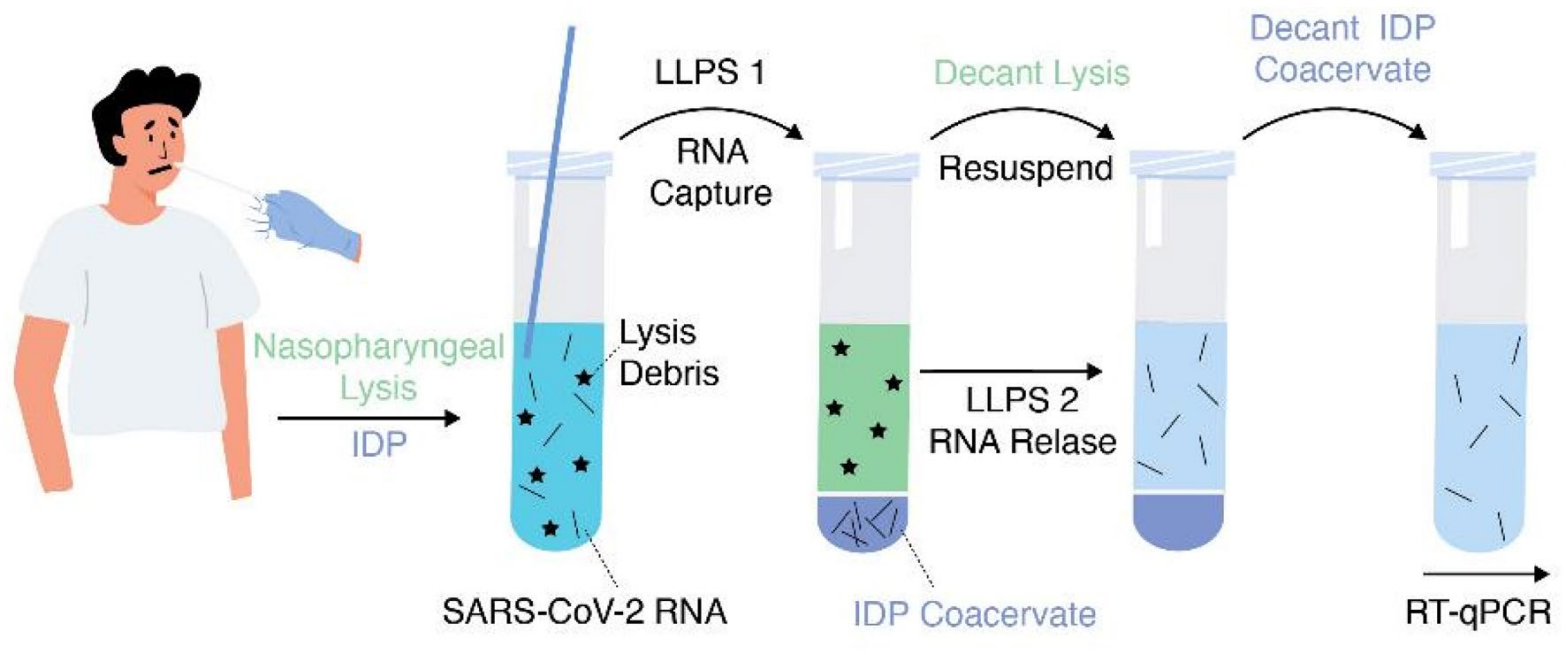
Schematic illustration of the two-step LLPS procedure for extraction of SARS-CoV-2 RNA from a nasopharyngeal swab sample prior to RT-qPCR.

This work examines the potential of NA-binding ELPs to extract NAs in clinical samples for downstream applications. First, we examine ELP-NA binding and dissociation with model DNA and RNA oligomers in artificial clinical-like solutions. For the experiments involving DNA, we use a 56 nt single-stranded oligonucleotide referred to as ssDNA1 (see Table S1 for sequence), and for those involving oligo-RNA, we use tRNA from baker’s yeast. Then, we examine extraction of inactivated viral RNA from SARS-CoV-2 by capture and release from ELP coacervates and determine extraction conditions compatible with downstream RT-qPCR (see Fig. 1), the current gold standard in viral NA detection. We chose SARS-CoV-2 RNA as a model NA analyte because most readers are now familiar with its detection as a diagnostic marker for COVID-19^29,30^, and because we had access to unidentified patient samples that contained this marker through UNM’s Center for Global Health. Optimization of such a simple method for NA extraction could have application in a wide range of molecular biological, diagnostic, and forensic applications.

## Results and discussion

### Binding of ELP fusion proteins and NAs in physiologically relevant fluids

**Figure 2:**
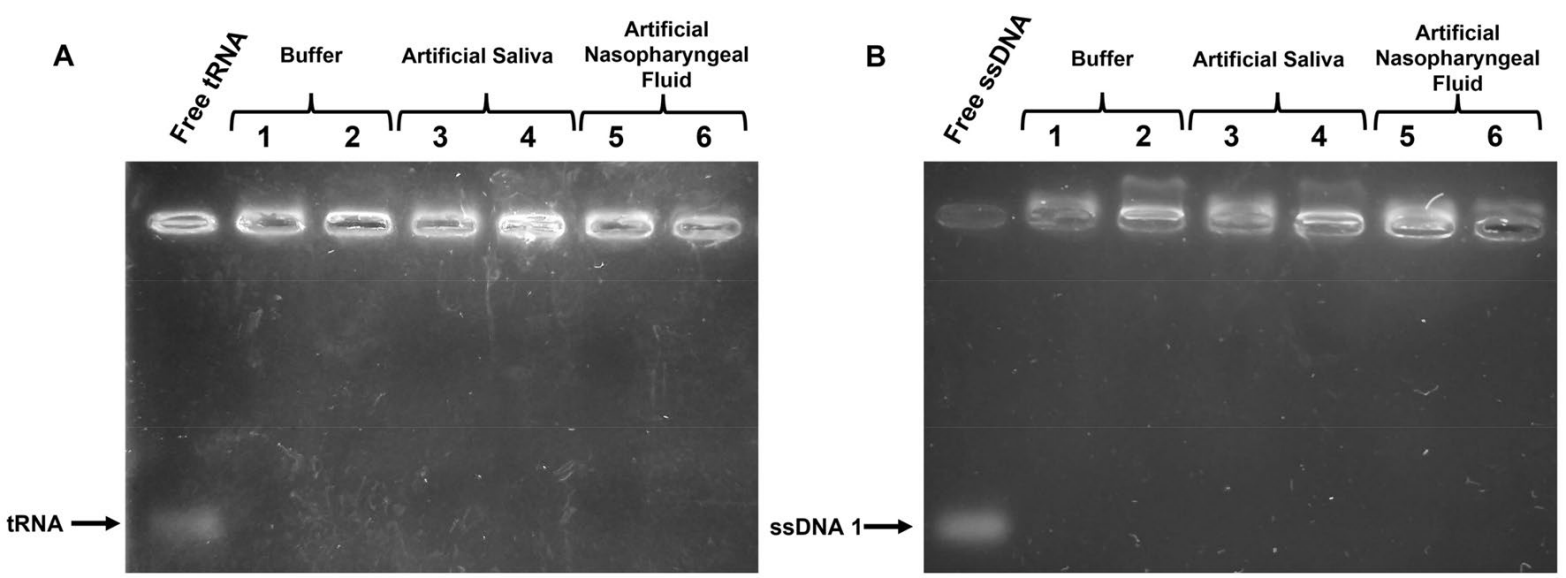
Gel retardation assays showing the binding of ELP fusion proteins and NAs in biologically relevant fluids. Agarose gels illustrate the binding activity of 0.1 mM E1.40COR30 (lanes 1, 3 and 5) and 0.1 mM E3.10 (lanes 2, 4, and 6) with either (A) 0.1 mg/mL tRNA or (B) 0.5 µM ssDNA1 in buffer (lanes 1 & 2), diluted artificial saliva solution (lanes 3 & 4), or diluted artificial nasopharyngeal fluid (lanes 5 & 6). Binding is indicated by retardation of NA electrophoresis.

We evaluated the NA binding activity of E3.10 and E140.COR30 after incubation at room temperature (below their coacervation transition temperature, T_t_) to ssDNA1 and tRNA in 3 different physiologically relevant solutions. Gel retardation assays reflect the binding capacity of the two ELP fusion proteins to ssDNA1 and tRNA in 1xPBS (Fig. 2A,B lanes 1–2), in 50% 1xPBS/50% artificial saliva (Fig. 2A,B, lanes 3–4) and in 50% 1xPBS/50% artificial nasopharyngeal swab fluid (Fig. 2A,B, lanes 5–6). The migration of both tRNA and ssDNA1 is significantly encumbered in the presence of the ELP fusion proteins, suggesting an interaction between the proteins and NAs in all three fluids. While the manufacturer (Biochemazon) of the artificial fluids does not disclose their composition, the product labels claim to simulate the mineral and salt composition, enzymes, pH, and viscosity of natural fluids. Published studies confirm that artificial saliva formulations often comprise salts such as sodium chloride or potassium chloride and other inorganic compounds^31^. Our results indicate an association between ELPs and NAs that is not disrupted by any component present in the complex artificial fluids. Both ELPs may have an affinity for structured nucleic acids such as tRNA and ssDNA1, which forms an intermolecular hairpin loop secondary structure^32–34^. Domains from FUS (i.e., RRM and RGG) are known to recognize RNA stem-loops and other DNA structures, and HCV CP is a known binder of the ssDNA1 in this study^35–38^. The flexibility and dynamics of the structure of IDPs has been previously identified as one of the reasons for binding secondary and tertiary structures of NAs^39,40^.

The demonstration that E140.COR30 and E3.10 bind NAs in solution (Fig. 2) and the high fidelity of temperature-controlled ELP phase separation motivated our exploration of the recruitment of NAs into coacervating ELP fusion proteins. These could then form nucleoprotein (NP) condensates as a platform for NA isolation and separation in the three biologically relevant solutions described above.

## ELP fusion proteins recruit ssDNA1, tRNA, and SARS-CoV-2 RNA into coacervates upon LLPS

**Figure 3:**
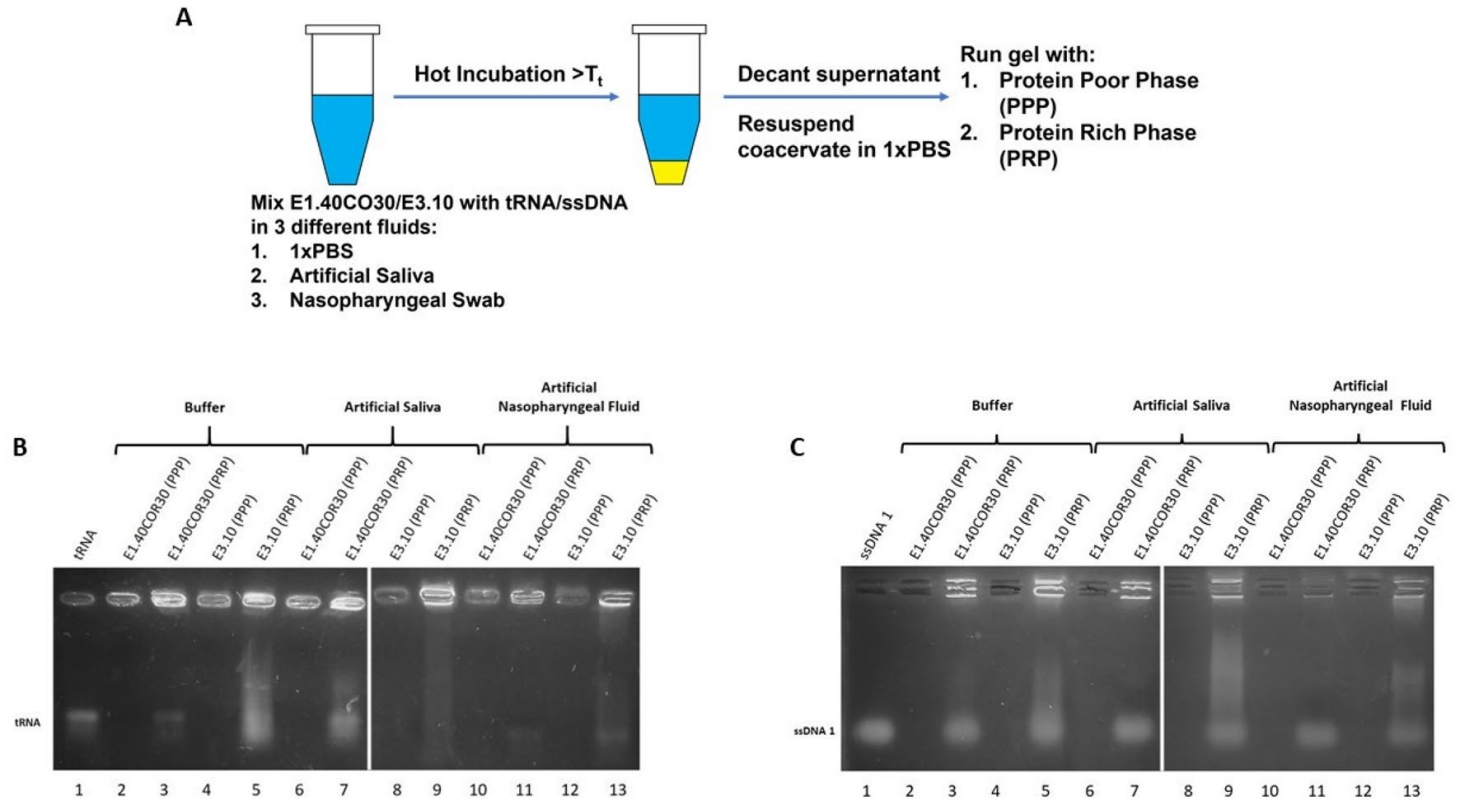
Recruitment of ssDNA1 and tRNA into ELP coacervates upon LLPS at 50 °C in different biologically relevant fluids. (A) Illustration depicting the workflow followed to examine recruitment of NAs into ELP coacervates upon LLPS in different media. To liberate the NAs from the ELPs prior to running the gels samples were incubated in a stopping buffer. Agarose gels illustrate the recruitment of (B) 0.1 mg/ml tRNA and (C) 0.5 µM ssDNA1 RNA into the protein rich phase (PRP) of 0.1 mM E1.40COR30 and 0.1 mM E3.10 upon LLPS. Smearing in gel lanes may be due to NA degradation or transient protein association.

Solutions of ELPs and NAs in each physiologically relevant fluid were prepared, and the workflow of the experiment is described in Fig. 3A. We note that the ELPs maintain their temperature-triggered phase behavior in artificial saliva and nasopharyngeal fluid. To our knowledge, LLPS of ELPs in these fluids has not been studied previously. Qualitative examination of the presence of NAs in both the protein-rich and protein-poor liquid phases after ELP coacervation was performed by collecting samples of each phase, disrupting ELP-NA binding by dilution with a “stopping buffer”, and subjecting the solutes to NA gel electrophoresis.

Inspection of the agarose gels shown in Fig. 3B,C suggests that ssDNA1 and tRNA are predominantly localized in ELP fusion protein-rich coacervate phases (lanes 3,5,8,10,13), indicating that, upon LLPS in all three media, both fusion proteins recruit tRNA and ssDNA1 into the condensed phase coacervates. Previous studies have already confirmed that ELPs and ELP fusions can recruit NAs nonspecifically upon LLPS and that RRM and RGG are involved in forming phase-separated NA-rich condensates^19,23,41,42^. Although RRM-RGG and HCV CP may bind different types of NA without any specific nucleotide preference, they tend to have some structural preference for complex structures such as hairpins or G-quadruplex^43,44^. As mentioned previously, the structural properties of IDPs allow binding to NAs with complex structures^43,44^.

These results framed the next phase of this study to examine (i) the binding and recruitment of viral RNA into ELP fusion condensates, and (ii) the potential utility of using NA-binding ELPs as reagents for RNA isolation from clinical samples for subsequent amplification and detection techniques such as PCR. Initially, we used purified clinical SARS-CoV-2 RNA (1 × 10^7^ copies) to examine the capture of viral RNA into ELP coacervates upon LLPS (Supplementary Fig. S1). By comparison to a standard curve of SARS-CoV-2 RNA dilutions (Supplementary Fig. S1), our assay results confirmed that E3.10 and E1.40.COR30 could form NP condensates with viral RNA upon phase separation. A mixture of 0.5 mM E3 and 10 μM E3.10 (which induces a more complete LLPS than E3.10 alone (i.e., complete phase coalescence); Figs. S3, S5) sequesters, and concentrates almost 10^5^ copies of viral RNA from solution, while 0.5 mM E1.40.COR40 concentrates almost 10^6^ viral RNA copies. However, to use these proteins as tools for isolation and assay, it is necessary to deactivate ELP fusion protein-RNA interactions because we found that the presence of ELPs in an RT-qPCR assay interferes with the amplification process (Supplementary Fig. S1). Further work will be required to identify suitable methods to disrupt E1.40.COR30-RNA and E3.10-RNA interactions. Instead, in the remainder of this study, we turned to experimentation with other ELPs in which interactions with NAs can be turned on and off by a pH switch.

## Phase behavior of pH-responsive ELPs in the presence of NAs

**Figure 4:**
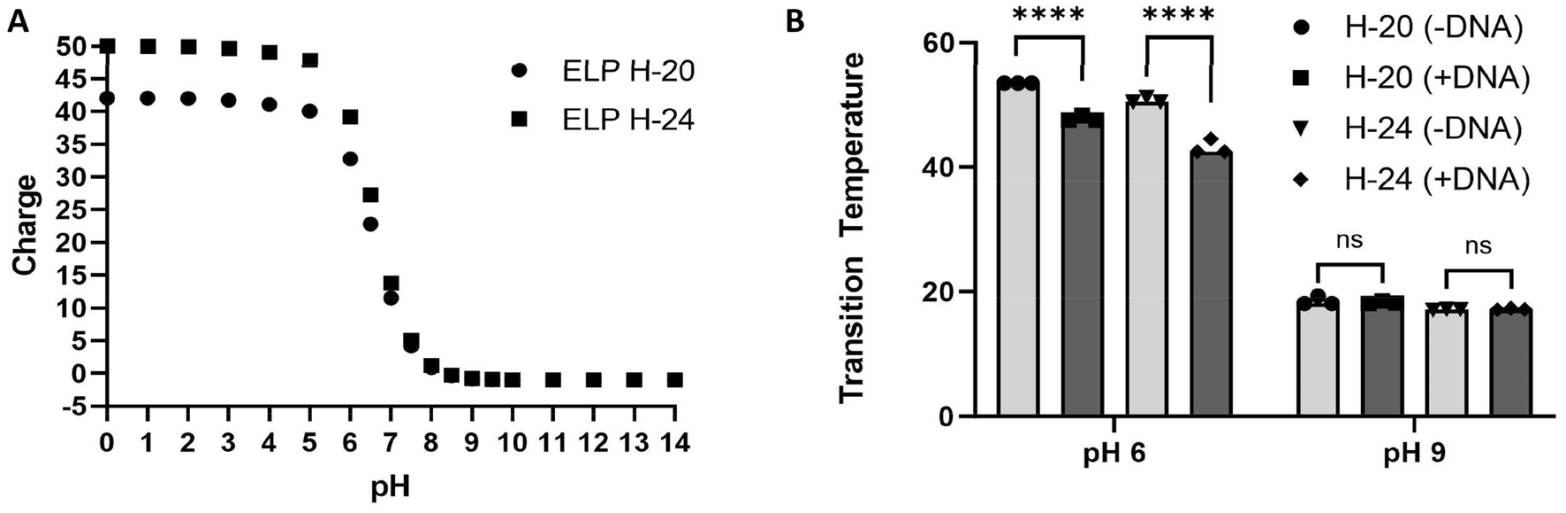
pH-sensitive charge and LCST behavior of H-20 and H-24. (A) Estimation by SnapGene of molecular charge vs pH for H-20 and H-24. (B) Characterization of the cloud point transition temperature (T_t_) for LLPS of 0.5 mM H-20 and H-24. T_t_ was measured in the absence and presence of 0.5 μM ssDNA1 in pH 6 buffer (37 mM citric acid/126 mM Na_2_HPO_4_) and pH 9 buffer (100 mM Tris). ****: *p* < 0.0001; *ns*: not significant.

MacKay and coworkers showed that H-20 and H-24 display pH-dependent LLPS, with T_t_ at 100 μM varying in an sigmoidal fashion from ≈75 °C at pH 5.5 to ≈25 °C at pH 8^24^. Figure 4A shows the charge on H-20 and H24 as a function of pH as estimated by SnapGene Viewer (SnapGene software; www.snapgene.com) and Fig. 4B presents our measurement of T_t_ at pH 6 and pH 9 as measured by turbidimetry (see Supplementary Fig. S6 for raw data). At pH 6, where we expect that each His-ELP will have ~ 30–40 positive charges, we observe a significant decrease of the T_t_ for H-20 and H-24 in the presence of a ssDNA1 (Figs. 4B, S6A,B), while the T_t_ is not altered by the presence of ssDNA1 at pH 9 (Fig. 4B, S6D and S6C), where we expect the His-ELPs to be close to neutral. As observed previously, NAs can alter the phase behavior of NA-binding ELPs^19,23^. This suggests that at pH 6, the His-ELPs bind the ssDNA1, while at pH 9, they do not. This pH-responsive binding behavior may be exploited to enable extraction of NAs upon LLPS. That is, we hypothesized that we can change aqueous solution conditions to weaken or strengthen the association between negatively-charged molecules, such as DNA or RNA, and His-ELPs, to create an on/off switch for protein-NA interactions. We thus explored the possible use of simple mechanisms of pH-dependent NA-binding and temperature and pH dependent LLPS of His-ELPs in the task of NA isolation.

## Electrostatic binding activity of His-ELPs to NAs at pH 6

**Figure 5:**
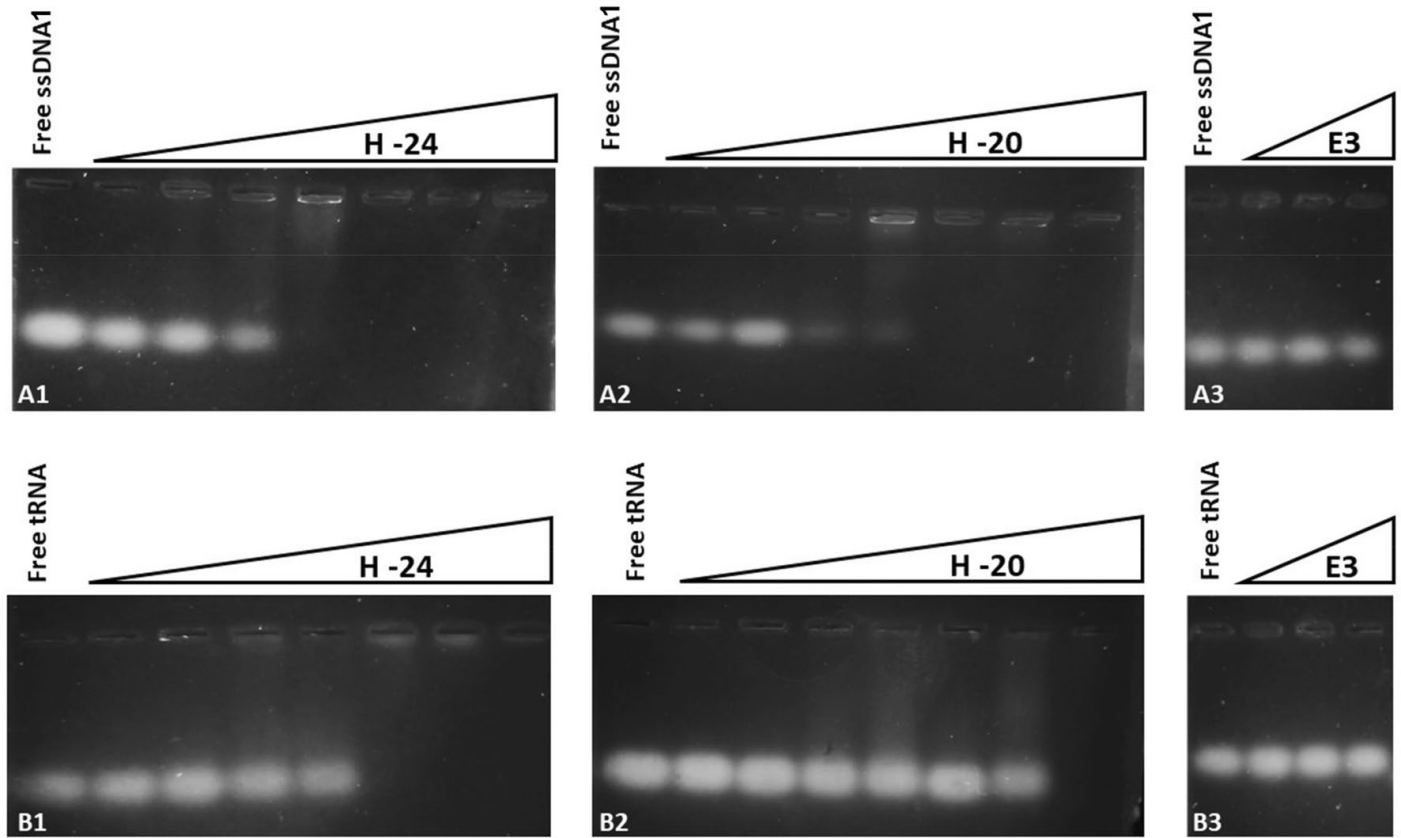
Gel retardation assays showing the binding of His-ELPs and NAs at pH 6. Agarose gels illustrate the concentration dependent binding activity of H-20 and H-24, but not of E3 with (A) 0.5 μM ssDNA1 and (B) 0.5 mg/ml tRNA in 37 mM citric acid/126 mM Na_2_HPO_4_ buffer at pH 6. (H-20 and H-24 concentrations are: 0.1, 1, 5, 10, 25, 50 and 100 μM. E3 concentrations are: 10, 100, 1000 μM).

According to the prediction of His-ELP charge as a function of pH (Fig. 4A), at pH 6, H-20 should have a charge of ≈ + 33 and H-24 a greater charge of ≈ + 39 as it has more histidines in its sequence. We hypothesized that the primary association between His-ELPs and NAs is via electrostatic interactions; thus, the protein with greater charge should have a more robust binding activity. We performed gel retardation assays to analyze the binding activity of the His-ELPs (and another ELP with 8 cationic lysine charges, E3) with tRNA and ssDNA1 as a function of protein concentration at pH 6. Binding and gel electrophoresis were conducted at room temperature, below the T_t_ of each of the proteins, and for both tRNA and ssDNA1, we observe that the degree of NA migration retardation is dependent on protein concentration (see Fig. 5). Moreover, the more charged protein polymer, H-24, retards migration of both NA species at lower concentrations, substantiating our hypothesis that the His-ELP with higher charge exhibits a stronger binding. We also observe that E3, which has only eight positively charged lysines, does not appreciably bind nucleic acids below its T_t_ (Fig. 5A3,B3). We thus conclude that at pH 6, both His-ELPs exhibit NA-binding activity in their soluble state.

**Figure 6:**
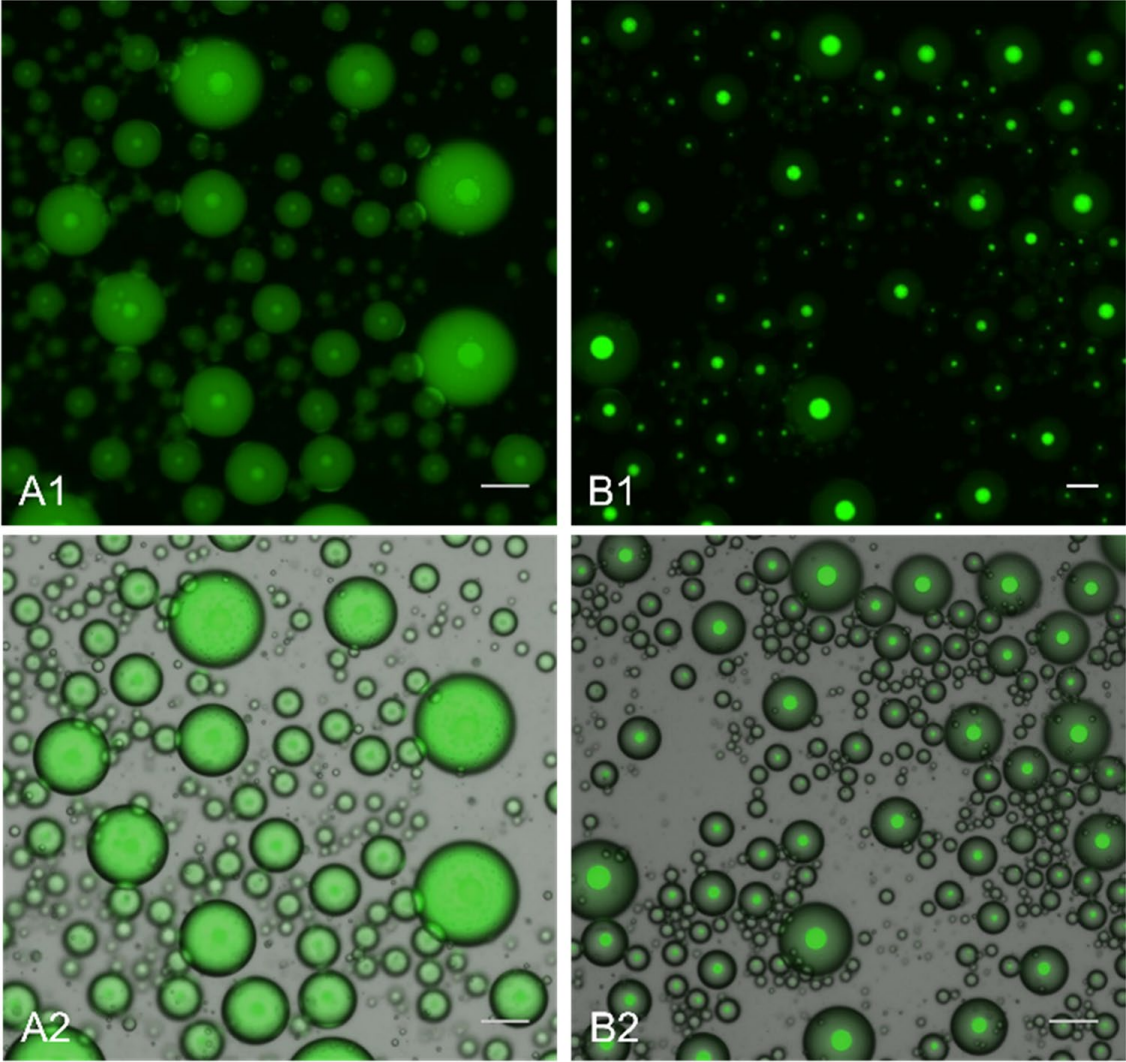
Fluorescence and brightfield microscopy of phase-separated aqueous microdrops in oil containing 0.5 mM H-24 and 0.5 μM ATTO488-labeled ssDNA1. (A, left) pH 8 (100 mM Na_2_HPO_4_/300 mM NaCl); (B, right) pH 6 (37 mM citric acid/126 mM Na_2_HPO_4_). Fluorescence microscopy (top: A1,B1) and bright field-fluorescence overlay (bottom: A2,B2). Images are taken after 20 min at 62 °C in a fully phase-separated state. Scale bars = 50 μm.

Using fluorescence microscopy, we confirmed that in pH 6 buffer, ssDNA1 is recruited into H-24 coacervates upon LLPS at 60 °C (Fig. 6). We imaged polydisperse water-in-oil droplets comprising H-24 and ATTO488-labeled ssDNA1 in 37 mM citric acid and 126 mM Na_2_HPO_4_ buffer at pH 6 after 20 min of hot (60 °C) incubation. We observed that most of the fluorescence from the labeled ssDNA1 is localized in the phase-separated protein condensates, indicating efficient capture of the DNA in the coacervate. Lysines and arginine have been widely studied as primary contributors to interactions between cationic residues and the polyanionic NA backbone^45–47^, but histidines can also be used to control NA-protein interactions in pH-and salt-dependent binding^46,48^.

## Reduced electrostatic binding of His-ELPs to NAs at higher pH

**Figure 7:**
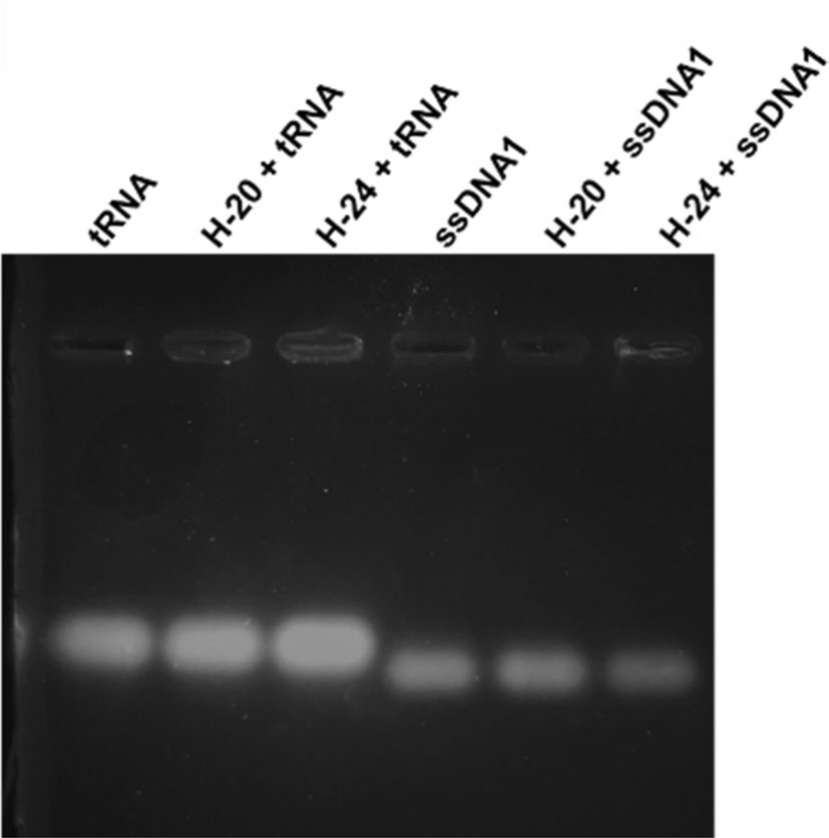
Gel retardation assays showing the lack of binding of His-ELPs at basic pH (4 °C). Agarose gels illustrate the absence of appreciable binding at 4 °C of 100 μM H-20 and H-24 with 0.5 mg/mL tRNA (lanes 1–3) and 0.5 μM ssDNA1 (lanes 4–6) in 100 mM Tris buffer at pH 9.

To demonstrate the modulation of electrostatic binding of His-ELPs and NAs, we performed gel retardation assay experiments with mixtures of His-ELPs and NAs at pH 9, where the proteins are predicted to be slightly negatively charged. To maintain the His-ELPs in their soluble state (below T_t_), binding and gel electrophoresis were conducted at 4 °C. As shown in the image of the gel presented in Fig. 7, the migration of tRNA and ssDNA1 was unaltered by the presence of His-ELPs at pH 9, confirming that in a higher pH environment, the neutralization of positive charges on the soluble His-ELPs (i.e., below their T_t_) decreases their association with NAs. A pH-induced change in charge can thus potentially be used as an on/off switch for His-ELP-NA interactions. However, pH 9 is not ideal for the stability of RNA, since exposure of RNA to highly alkaline solutions can lead to their degradation (hydrolysis)^49,50^. We consequently employed a less basic buffered solution at pH 8 to suppress associations between the His-ELPs and NAs. At pH 8, the His-ELPs are predicted to have a slight positive charge. We thus examined binding in a buffer at pH 8 with added salt (300 mM NaCl) for the following reasons: (i) NaCl will shield residual positive charges of the His-ELPs, and (ii) for isolation/extraction methods in coacervates that rely on the LLPS of ELPs, the addition of salt will reduce the transition temperature of His-ELP. A gel retardation assay showed that under these buffer and salt conditions, the migration of ssDNA1 and tRNA was not affected by the presence of soluble His-ELPs (i.e., below their T_t_; results not shown).

We used fluorescence microscopy to image polydisperse water-in-oil droplets comprising H-24 and ATTO488-ssDNA1 in 100 mM Na_2_HPO_4_ and 300 mM NaCl buffer at pH 8 after 20 min of incubation at 60 °C (i.e. above the T_t_ of H-24 at these conditions). As shown in Fig. 6, we observed that the fluorescence signal from the ssDNA1 across the aqueous microdroplets, showing little partitioning or preference for either of the two phases (i.e., protein-rich or protein-poor phases). These results are similar to those in previous studies with the charged ELP, E3, which showed that DNA-ELP interactions were deactivated upon charge shielding, and there was no preferential partitioning of DNA into E3 coacervates after phase separation^23^. Based on these results, at higher pH, neither H-20 nor H-24 show any evidence of NP formation either below or above their T_t_. Histidines have been previously used as pH switches for electrostatic interactions between protein-NA, peptide-ligand, and protein–protein interactions^51^. These results suggest a simple thermal and pH-responsive capture and release process for NAs in His-ELP coacervates upon LLPS in different solutions that does not require extensive equipment nor technical expertise. These results provide the bases for our implementation of these processes for isolation of NAs in more complex, physiologically and biomedically relevant fluids.

### tRNA and ssDNA1 can be extracted from physiologically relevant fluids with His-ELPs in two LLPS steps

**Figure 8:**
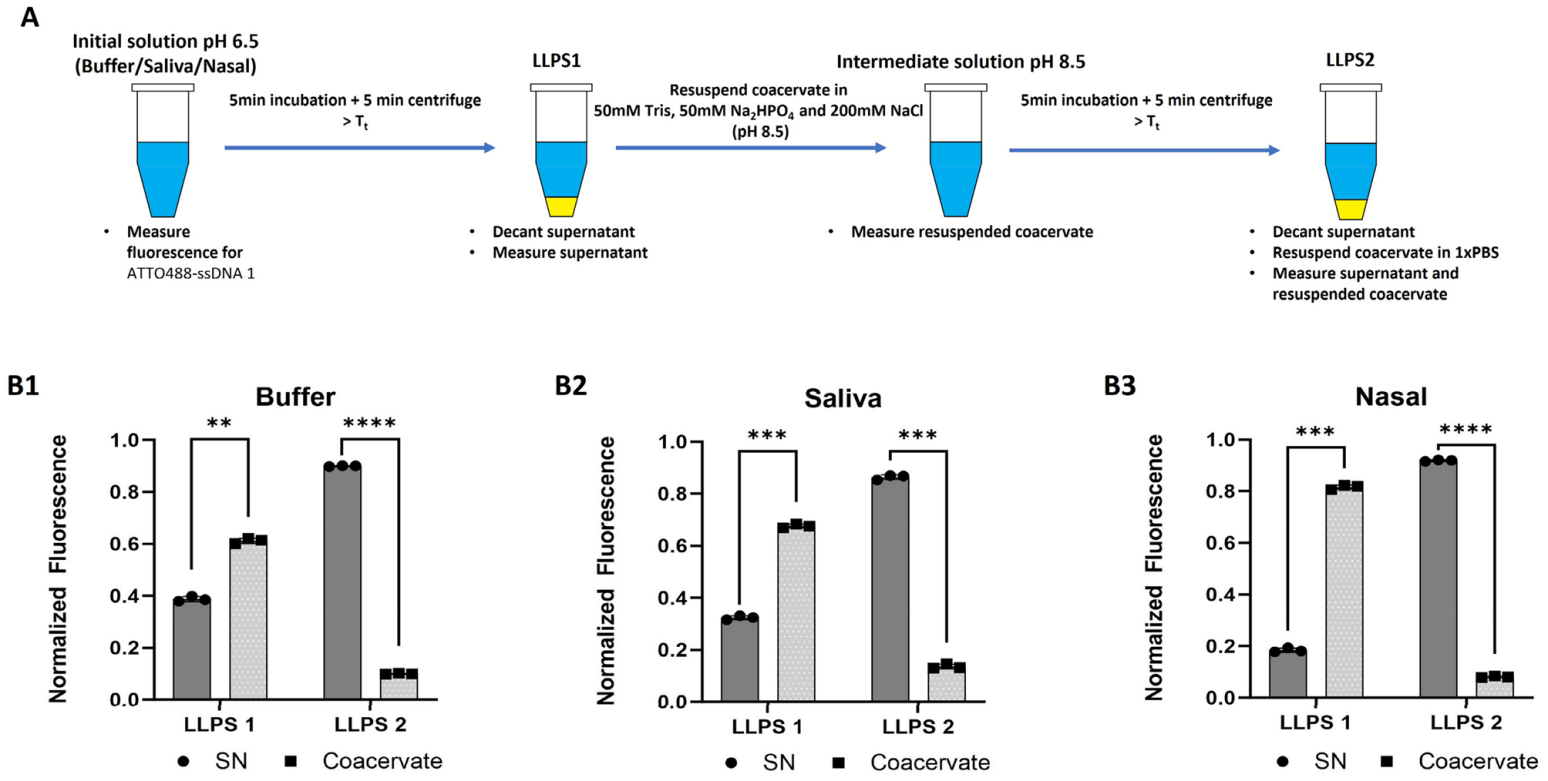
Recruitment of ssDNA1 into H-24 coacervate from different physiologically relevant solutions and subsequent release upon LLPS after pH shift. (A) Workflow of two-step NA isolation assay. (B) Fluorimetry measurements of an ATTO488-labeled ssDNA1 in the supernatant (SN, circles, dark gray bars) and coacervate (squares, light gray bars) were taken after LLPS 1 and LLPS 2 for the three physiologically relevant solutions. ****: *p* < 0.0001; ***: *p* < 0.001; **: *p* < 0.01.

We designed a two-step NA extraction process that takes advantage of the dual stimulus responsive behaviors of His-ELPs (pH-responsive NA binding and temperature triggered LLPS) to isolate tRNA and ssDNA1 from buffer, artificial saliva, and artificial nasopharyngeal swab solutions. The goal here is the recovery of a NA-rich solution free of significant amounts of contaminants. First, we performed a fluorescence assay to examine the partitioning of ATTO488-ssDNA1 after LLPS of positively charged H-24 in the three fluids (buffer, saliva, and nasal swab; Fig. 8). Briefly, we prepared a mixture of H-24 and ATTO488-ssDNA1 in the three different fluids at pH 6.5, and measured the initial fluorescence of each sample. Next, to induce phase separation, we incubated the solutions at 60 °C for 5 min and centrifuged them at 60 °C for 5 min. We carefully pipetted out the supernatant and resuspended the coacervate to the same initial volume with 50 mM Tris, 50 mM Na_2_HPO_4_ and 200 mM NaCl (pH 8.5) buffer and measured the fluorescence of the resuspended protein-rich coacervate as well as that of the protein-poor supernatant (SN). Consistent with our previous observations obtained by electrophoresis and fluorescence microscopy, Fig. 8B shows that at pH 6.5 (LLPS 1), H-24 binds and partitions ssDNA1 into NP condensates in buffer, artificial saliva, and artificial nasopharyngeal fluid.

Interestingly, the partitioning of ssDNA1 into H-24 coacervates at pH 6.5 (LLPS 1) seems to be more effective in the pseudo-clinical solutions than in buffer. While approximately 60% of the ssDNA1 is present in the buffer coacervate (Fig. 8B1), ≈70% is present in the coacervate formed in artificial saliva samples (Fig. 8B2), and ≈80% separates in the artificial nasal swab sample (Fig. 8B3). These results may reflect differences in solution conditions (e.g., ionic strength).

We redissolved the protein-rich phase by adding 100 mM Na_2_HPO_4_ / 300 mM NaCl buffer (pH 8) to the initial volume, and induced a second LLPS of H-24 for 5 min at 30 °C, followed by a 5-min room temperature centrifugation to induce coacervate coarsening. We then measured the fluorescence for the newly formed protein-poor and protein-rich phases, and we observed that over 90% of the fluorescent ssDNA1 was in the protein-poor supernatant after the second LLPS (Fig. 8B), indicating that the ssDNA1 did not partition into the ELP coacervate at pH 8. Our results are consistent with the results of fluorescence microscopy (Fig. 6) and the gel retardation assay (Fig. 7); the salt-rich buffer at pH 8 impedes NA-binding and allows recovery of the NA from H-24. As shown in Supplementary Fig. S4, at 0.5 µM ATTO488-ssDNA1, the intensity of fluorescence slightly increases with pH. Since the pH for LLPS2 is lower than the pH for LLPS1, the difference in fluorescence intensity between the LLPS1 samples and LLPS2 samples cannot be due to a pH difference.

We performed a similar NA isolation experiment replacing the ssDNA1 with tRNA (see Supplementary Fig. S2). Via standard agarose gels, tRNA traces were observed in the intermediate protein-rich solutions and the final protein-poor supernatant. No tRNA was observed in the intermediate protein-poor supernatant for buffer and artificial saliva samples, and some tRNA was observed in the artificial nasal swab sample. Moreover, we kept 15uL of the initial solutions, the intermediate solutions, and the “pure” supernatants after LLPS 2 and ran them on an SDS-PAGE gel to examine the extent of protein contamination after the two LLPS steps for each solution type. The SDS-PAGE gel showed no protein bands in the second supernatant, indicating that our method yielded a tRNA-rich solution with no significant protein contaminants from each physiologically relevant solution.

These results indicate that we successfully developed a process to isolate nucleic acids from buffer and complex clinical-like fluids. This method works efficiently for short ssDNA1 and tRNA without the need for expensive reagents, equipment, or technical expertise. Moreover, the reversible LLPS behavior of ELPs can be further exploited because one could potentially reuse the proteins for multiple extractions.

## Extraction of SARS-CoV-2 RNA from COVID patient samples using H-24 ELP

As a proof-of-concept demonstration of the utility of the reversible LLPS and NA-binding behavior of His-ELPs, we applied H-24 in the simple two-step extraction process described above to isolate SARS-CoV-2 RNA from inactivated VTM samples from nasopharyngeal swabs of de-identified human COVID-19 patients. The efficacy of our unoptimized His-ELP enabled extraction process was compared to that of a commercially available spin column methodology (Quick-RNA™ Viral Kit, Zymos Research) in the detection of SARS-CoV-2 RNA by RT-qPCR. The N1 primer/probe set used in the RT-qPCR experiments specifically amplifies a portion of the SARS-CoV-2 genome. Results of extraction of nasopharyngeal swab samples from a de-identified human COVID-positive patient were compared with those from a COVID-negative patient. Representative data from replicate measurements of each sample were made on different days and are provided below.

We subjected the nasopharyngeal swab samples (suspended in VTM and DNA/RNA Shield™) to cell lysis by heat shock and then mixed the lysate with ELP H-24 in a pH 6 solution. We then incubated the samples above the T_t_ of H-24 to induce LLPS with the objective of recruiting RNA into the protein coacervate phase to isolate it from the other lysate components. After phase separation, we carefully pipetted out the protein-poor supernatant and resuspended the coacervate in a pH 8.5 solution to disrupt electrostatic interactions between RNA and H-24. We then incubated the solution above the T_t_ of the neutralized H-24 to induce LLPS with the objective of separating the H-24 protein from the RNA. We carefully pipetted out the supernatant for PCR detection. Finally, we diluted supernatants in nuclease-free water, as final extraction products are usually eluted in water.

Using the commercially available RNA extraction kit, SARS-CoV-2 RNA was detected in the samples from the COVID-positive patient by RT-qPCR, with a cycle threshold (C_T_) value of 25. The C_T_ value refers to the number of cycles necessary for the RT-qPCR process to detect a specific RNA sequence. A smaller value is correlated with higher RNA concentration in a sample. No SARS-CoV-2 RNA was detected in the sample from the COVID-negative patient using the commercial RNA extraction kit. By comparison, after the two-step LLPS/pH switch process described above, RT-PCR did not detect SARS-CoV-2 RNA directly in the supernatant after the LLPS at pH 8.5, but it did detect it (C_T_ = 37) after the supernatant was diluted 1:50 with nuclease-free water. Interestingly, the target RNA was not detected after a similar 1:10, 1:20, nor 1:100 dilution, suggesting an optimal dilution, perhaps representing a balance of dilution of PCR inhibitors and sufficient SARS-CoV-2 RNA concentration for detection. The higher C_T_ value obtained for the LLPS-based extraction suggests lower efficiency than the conventional extraction in this preliminary, proof-of-concept experiment. To examine whether the initial LLPS step at pH6 resulted in incomplete capture of SARS-CoV-2 RNA into the coacervate phase, we conducted RT-qPCR on the supernatant obtained from that initial LLPS step. While detection of the target RNA was not possible directly in the supernatant, RNA was detected when this supernatant was diluted with nuclease-free water (1:10 C_T_ = 39; 1:20 C_T_ = 37; 1:50 C_T_ = 36; 1:100 not detected), with optimal detection (lowest C_T_) at 1:50 dilution. No SARS-CoV-2 RNA was detected in the sample from the COVID-negative patient using the LLPS-based extraction under all dilution conditions studied.

These results demonstrate that the two-step H-24 LLPS process with pH shift is able to extract SARS-CoV-2 RNA from patient samples for detection by RT-qPCR, albeit after significant dilution in nuclease-free water and at higher C_T_ than the standard commercial method. Several factors may need optimization including the amount of H-24 used in the extraction process, the time to achieve LLPS, and the solution conditions for LLPS. Also, the simple lysis procedure used here (heat shock) may not be optimum. The results of this initial investigation provide significant impetus for further study. The observation that RT-qPCR was capable of detecting SARS-CoV-2 RNA after one round of LLPS and some dilution, suggests that the first LLPS process was not sufficient for capturing all SARS-CoV-2 RNA. Further, the observation of an optimum dilution for RT-PCR detection suggests that competing factors (e.g., related to concentrations of RNA and inhibitors of the NA detection processes) are at play.

## Conclusion

This work explores a new method for isolating nucleic acids from biomedically relevant solutions using engineered smart nanomaterials. We engineered several NA-binding IDPs capable of undergoing temperature triggered LLPS in different physiologically relevant fluids to recruit NAs into NP coacervates nonspecifically. Upon exploring different approaches, we developed a methodology based on the pH-dependent charge of histidine to engineer ELPs that are capable of binding and releasing NAs upon a simple pH switch. At pH 6.5, His-ELPs proved to be robust NA-binders in solution below their Tt and to recruit NAs upon LLPS when heated above their T_t_ to form NP condensates. A change in pH completely alters the binding capacity of the proteins. Above pH 8, NA-binding is deactivated, both below and above T_t_, such previously bound NAs can be released and LLPS of the ELP can be used to remove it from the NA-containing solution. ELPs are an attractive choice of polymers for such functions because their temperature and pH response can be easily tuned. They are readily expressed in standard *E. coli* bioreactors and easily purified by inverse transition cycling. This study has demonstrated that the dual responsive nature of His-ELPs –in which LLPS and NA-capture are sensitive to temperature and pH– provides unique function to enable facile NA extraction and release from complex samples and thus new sample preparation methodologies for use in NA-based detection of pathogens or other NA-based analytical methods.

This study explored the phase behavior and NA sequestration/release in four fluids: buffer, artificial saliva, artificial nasopharyngeal swab solution and human nasopharyngeal swab samples. Our aim was to establish a proof-of-concept demonstration of the potential suitability of a new methodology for sample preparation of patient samples for molecular diagnostics of viral RNA, but this approach may be extended to other NA analytes and other fluids such as urine, plasma, blood, or even sewage or other complex, non-physiological solutions. Recombinantly expressed, stimuli responsive IDPs such as the ones presented in this study are excellent model polymer systems with precisely defined sequence and molecular weight. As suggested by this study, they can be useful in providing design criteria for the synthesis of non-peptide polymers that are temperature and pH responsive, but that may be more amenable to scaled-up manufacture, for the extraction of NAs by LLPS. We envision a wide range of applications of this methodology in applications from molecular diagnostics to forensics, in each case without the need for expensive equipment or advanced technical skills. Table S2 presents a comparison of the likely potential features of an optimized methodology for NA extraction using LLPS in relation to those of several commonly employed methods.

## Methods

### Materials

The expression vector pET24 was purchased from Novagen, Inc. (Milwaukee, WI). One-Shot BL21 Star (DE3) *Escherichia coli* cells were from ThermoFisher Scientific (Waltham, MA). Restriction enzymes were from New England Biolabs (Beverly, MA). DNA purification kits were purchased from QIAGEN, Inc. (Valencia, CA). DNA sequences (genes fragments and ssDNA1) were purchased from Integrated DNA Technologies (Coralville, IA). tRNA was purchased from Millipore Sigma (St. Louis, MO). Luria broth (LB) agar plates were purchased from Bacto Agar, Becton Dickinson (Franklin Lakes, NJ), and Millipore Sigma (St. Louis, MO). Kanamycin was from Ultrapure, VWR, (Radnor, PA). LB Broth and Terrific Broth (TB) was from IBI Scientific (Dubuque, Iowa). Viral RNA isolation kit, was from Zymo Research (Irvine, CA). Reagents for RT- qPCR were obtained from ThermoFisher Scientific (Waltham, MA).

### Plasmid construction

The gene encoding the E3.10 protein was constructed using plasmid pET24-E3 as a starting point^24^. The RRM and RGG domains of the FUS proteins are engineered into the E3 protein using the Golden Gate assembly method as described^52^. Briefly, the E3 plasmid and the FUS protein plasmid was digested with BsaI, and subsequently ligated together to generate pET24-E3.10. pET24-E1.40COR30 was constructed by ligating a synthetic COR30 sequence (Integrated DNA Technologies, Coralville, IA) to the 3’ end of the E1.40 sequence in pET24-E1.40 using a single step recursive ligation method^53^. Plasmids expressing H-20 and H-24 were constructed following previously described methods^28^.

### Expression and purification of ELPs and ELP fusion proteins

*Escherichia coli* (BL21) cells harboring plasmids encoding the protein of interest were inoculated onto LB agar plate containing 45 μg/mL kanamycin sulfate and incubated overnight at 37 °C. Starter cultures grown from individual colonies were used to inoculate 3 mL of LB broth with 45 μg/mL kanamycin sulfate. This culture was incubated overnight at 220 rpm and 37 °C. The culture was then transferred into 1L of TB supplemented with 45 μg/mL kanamycin sulfate. Cultures were incubated at 37 °C with agitation for 6 h before induction with 1 mM isopropyl β-d-1-thiogalactopyranoside (IPTG). The culture was induced at 37 °C for 18 h prior to harvest by centrifugation at 4 °C and 3000 rpm for 30 min. The resulting pellets were resuspended into a lysis buffer (phosphate buffered saline (1X PBS), 1 Pierce™ protease inhibitor tablet from ThermoFisher (Waltham, MA), and 0.05 mM ethylenediamine tetraacetic acid (EDTA) at pH 8.0) and lysed by sonication to release all intracellular content.

Expressed proteins were purified by inverse transition cycling, exploiting the reversible thermally responsive protein phase separation inherent to ELP constructs^54^. This approach consists of cyclic centrifugation steps that alternate between cold (4 °C) and hot (40 °C) centrifugation in PBS until all contaminants are removed, usually within 2–5 cycles. For E3.10, hot centrifugation was replaced by room temperature centrifugation. Ammonium sulfate (1 M) was added to induce ELP phase separation at room temperature and to avoid possible denaturation of folded domains in the protein^55^.

### Characterization of NA-ELP binding at room temperature by gel retardation assay

Solutions of 100 µM ELP mixed with 0.5 µM of ssDNA1 or 0.1 µM of tRNA were tested to ascertain ELP binding ability. Protein and NA concentrations and buffer compositions used are described in Fig. 2. Each ELP and NA combination used was incubated for 30 min at room temperature and subsequently loaded into 2.5% agarose gels for electrophoresis. To liberate the NAs from the ELPs prior to running the gels, samples were incubated in a stopping buffer. Gels are run at 90 V for 60 min at 4 °C, post-stained with SyBr Gold, and imaged in a transilluminator. Gels loaded with E1.40COR30, E3, and E3.10 were made and run with a 1X TBE buffer. Binding reactions for His-ELPs and NAs were run below and above their isoelectric point (estimated to be pH≈8.3). Positively charged His-ELPs were reacted with NA at pH 6 (37 mM citric acid and 126 mM Na_2_HPO_4_) and pH 8 (100 mM Na_2_HPO_4_). Gels for pH 6 and pH 8 His-ELP reactions were assembled and run with sodium hydroxide/sodium dihydrogen phosphate buffer at pH 7^56^. Negatively charged His-ELPs (pH 9) were run in Tris and NaCl buffer (100 mM Tris, 250 mM NaCl). Gels for pH 9 His-ELP / NA reactions were assembled and run with sodium carbonate /sodium bicarbonate buffer at pH 11 ^56^.

### NA recruitment into ELP coacervates upon LLPS

Solutions of 100 µM ELP mixed with 0.1 µM of tRNA were prepared. Each solution was incubated at 60 °C for 5 min and centrifuged at 60 °C for 5 min to induce LLPS. After LLPS, the protein-poor phase (supernatant) was decanted, and the protein-rich phase (coacervate) was resuspended in 100 μL PBS. To facilitate NA migration into the gel, a stopping buffer (20% glycerol, 20 mM EDTA pH 8.0, 2%SDS, 0.25% bromophenol blue) was used to denature the ELPs and disrupt NA-protein interactions^57^. Denatured samples (10 μL) of the protein-poor-phase and the protein-rich phase were separated and visualized on 2.5% agarose gels as described above.

### Recruitment of viral RNA in ELP coacervates

Phase separation of E3.10 upon thermal stimulus differs from simple ELPs, such as E3, because LLPS is not reversible and it does not form fully coalesced coacervates without centrifugation (Supplementary Fig. S3). The addition of E3 to E3.10 can help achieve a more fully coalesced coacervate (Supplementary Fig. S3).

Purified SARS-CoV-2 RNA (10^7^ copies, isolated from SARS-CoV-2 WA1-USA strain, BEI Resources) was mixed with either (i) 0.5 mM E1.40COR30, or (ii) a mixture 0.5 mM E3 + 0.1 mM E3.10 in viral transport media (VTM: 1 × Hanks buffered salt solution, 2% heat-inactivated fetal bovine serum, 100 μg/ml gentamycin, 0.5 μg/ml amphotericin B). The mixture was incubated at 50 °C for 10 min, followed by centrifugation for 3 min at room temperature to induce separated phases and coalescence. The protein-poor supernatant was collected into a different microfuge tube. The protein-rich coacervate was resuspended with VTM. The Quick-RNA™ Viral Kit (Zymo Research) was utilized to recover all pulled-down viral RNA from the protein-rich and protein-poor fractions after LLPS. Recovered viral RNA was quantitated by RT-qPCR using the N1 primer/probe set (CDC 2019-nCoV Real-Time RT-PCR diagnostic panel) on a StepOnePlus Real-Time PCR instrument (Applied Biosystems) (Supplementary Fig. S1). Similar experiments were performed in the absence of viral RNA recovery with the commercial kit. (Supplementary Fig. S1).

### Characterization of cloud point (coacervation) temperature (T_t_) of His-ELPs in the presence of ssDNA1 at different pHs by turbidimetry

Samples containing 0.5 mM of H-24 or H-20 in the presence or absence of ssDNA1 were prepared in 37 mM citric acid and 126 mM Na_2_HPO_4_ (pH 6) buffer, or 100 mM Tris (pH 9) buffer. The T_t_ was obtained by measuring the absorbance of the samples at 380 nm as a function of temperature and pH in a temperature-controlled UV–vis spectrophotometer (Cary 300 UV − vis, Agilent) as previously described^46^. The T_t_ was obtained by taking the maximum in the first derivative of the absorbance as a function of temperature of triplicate samples.

### Two-step isolation of model NAs from physiologically relevant solutions using ELP H-24

A two-step, NA isolation protocol was designed to study the separation of model NAs from other components present in model sample solutions using two LLPS cycles of ELP H-24 (Figs. 7A and S2). Two sets of 0.5 mM ELP-NA solutions (100μL each) were prepared, one with 0.2 mg/ml tRNA and the other with 0.5 µM ATTO488 labeled ssDNA1. The first step of the experiment consisted of preparing a mixture of protein and NA in (i) 1xPBS, (ii) 50% 100 mM Na_2_HPO_4_ / 50% artificial saliva, and (iii) 50% 100 mM Na_2_HPO_4_ / artificial nasopharyngeal swab solution at pH 6.5. Phase separation was induced at 65 °C for 5 min, followed by a 65 °C hot centrifugation for 5 min to achieve a clear phase separation. After two distinct phases were formed, the protein-poor phase (supernatant) was removed, while the protein-rich phase (coacervate) was resuspended to the initial volume with in pH 8.5 buffer (50 mM Tris, 50 mM Na_2_HPO_4_ and 200 mM NaCl). This buffer reduces the nominal positive charge from 27 to − 1 (SnapGene Software https://www.snapgene.com/; see Fig. 4); NaCl was included to promote the second phase separation. The second LLPS of the solubilized protein-rich phase was induced at 30 °C for 5 min, followed by a room temperature centrifugation for another 5 min. The supernatant again was recovered, while the coacervate was resuspeded in 1xPBS. To quantify the separation of ssDNA1, the fluorescence signal emitted by the ATTO488 labeled oligo in the protein-poor and protein-rich phases after each step of LLPS was measured at ex/em 488/520 nm using a microplate reader (Biotek Synergy H1; Agilent, Santa Clara, CA). The fluorescence after LLPS1 was normalized by dividing the fluorescence of the supernatant and the coacervate by the initial fluorescence. The fluorescence after LLPS2 was normalized by dividing the fluorescence of the supernatant and the coacervate by the intermediate fluorescence. As a control, we examined the influence of pH on the fluorescence of ATTO488 ssDNA1 by measuring the emission of ATTO488 at 3 different concentrations at 3 different pHs (Supplementary Fig. S4). In each case, fluorescence measurements were taken on triplicate samples. To study the partition of tRNA upon LLPS, 10µL of each protein-poor and protein-rich phase were separated on a 2.5% agarose gel as described above. Aliquots of the resuspended protein-rich phases were checked by SDS-PAGE for the presence of protein contaminants (Supplementary Fig. S2).

### Two-step extraction of SARS-CoV-2 RNA from patient samples by LLPS of H-24

Nasopharyngeal samples from deidentified COVID-19-positive patients were utilized to test the efficacy of H-24. These samples were previously inactivated in DNA/RNA Shield ™ (Zymo Research) after they were collected from consented patients who were part of another study (UNM Health Sciences Center Human Research Protection Office, protocol #20–680). SARS-CoV-2 RNA was previously isolated from the selected patient samples using the Quick-RNA™ Viral Kit (Zymo Research) and quantitated by RT-qPCR as described above. For these experiments, inactivated VTM was heated at 95 °C for 10 min to induced cell lysis^58^. For the first pull-down, an aliquot (200μL) of the lysed cells was mixed with 0.5 mM of H-24 in a pH 6 solution in a final volume of 800μL. The mixture was incubated at 65 °C for 10 min followed by centrifugation (90 s) at room temperature to induce coalescence. The resulting protein-poor supernatant was transferred to another tube. The remaining coacervate was resuspended in 100μL of a pH 8.5 buffer (50 mM Tris, 50 mM Na_2_HPO_4_ and 200 mM NaCl), and the incubation step was repeated in a second extraction step. Following centrifugation, the second protein-poor phase was transferred to another tube. Both protein-poor phases were diluted in H_2_O prior to RT-qPCR with the N1 primer/probe sets. Control experiments were performed using nasopharyngeal samples from COVID-negative patients.

### Supplementary Information

Supplementary Information.

## Data Availability

All data generated or analyzed during this study are included in this published article and its Supplementary Information file.
