# Supplementary material for: Isolation of nucleic acids using liquid–liquid phase separation of pH-sensitive elastin-like polypeptides
Source: Sci Rep. 2024 May 2;14:10157. doi: 10.1038/s41598-024-60648-9 (PMC11065875; doi:10.1038/s41598-024-60648-9)
Supplement: Supplementary file 1 — Supplementary Information. [file 41598_2024_60648_MOESM1_ESM.docx]

# SUPPLEMENTARY INFORMATION FOR:

# Isolation of nucleic acids using liquid-liquid phase separation of pH-sensitive elastin-like polypeptides

Telmo Díez Pérez,^1,2,3^ Ashley N. Tafoya,^1,2,3^ David S. Peabody,^4^ Matthew R. Lakin,^1,3,5^ Ivy Hurwitz,^6^ Nick J. Carroll,^2,3^ and Gabriel P. López^1,2,3,*^

^1^Center for Biomedical Engineering, ^2^Center for Micro-Engineered Materials, ^3^Department of Chemical and Biological Engineering, ^4^Department of Molecular Genetics and Microbiology, ^5^Department of Computer Science, University of New Mexico, Albuquerque, NM, 87131, USA, ^6^ Center for Global Health, Department of Internal Medicine, University of New Mexico Health Sciences Center, Albuquerque, NM 87131, USA

^*^Corresponding author. Email: [gplopez@unm.edu](mailto:gplopez@unm.edu)

| **DNA** | **Sequence (5’-3’)** | **Type** | **Length (nt)** |
| --- | --- | --- | --- |
| ssDNA1 | GGGTTCCTTGCTAGCCAGAGAGCTCCCGGGCTCGACCTGGTCTAACAAGAGAGACC | Single Stranded | 56 |
| ATTO488-ssDNA1 | GGGTTCCTTGCTAGCCAGAGAGCTCCCGGGCTCGACCTGGTCTAACAAGAGAGACC-ATTO488 | Single Stranded | 56 |

Table S1: Description of DNA oligomers used in experiments.

| Method | Principle | Advantages | Disadvantages | Equipment/Reagents | Price | Time (mins) | Experienced staff required |
| --- | --- | --- | --- | --- | --- | --- | --- |
| Phenol-Chloroform [1] | Separation via organic solvents | High yield, preserves RNA integrity | Toxic chemicals, labor intensive | Centrifuge, organic solvents, phenol, chloroform | Moderate: Readily available chemicals and no proprietary reagents | 60-90 | Yes |
| Guanidinium Thiocyanate (Trizol) [2], [3] | Disruption of cells with guanadine thyocyanate followed by organic extraction | High yield, compatible with various samples types | Time-consuming, requires careful handling of hazardous materials | Centrifuge, homogenizer, guanidine thiocyanate, chloroform, isopropanol | Moderate: Proprietary reagents may be more expensive than traditional methods | 50-70 | Yes |
| Silica-based columns [4], [5] | Binding pf RNA to silica membrane followed by wash and elution steps | High purity, compatible with automation | Yield may vary, expensive consumables | Vacuum manifold, centrifuge, silica-based columns | Moderate: Columns cost is variable between manufacturers | 40-60 | No |
| Magnetic Beads [6],[7] | Magnetic particles bind RNA, followed by wash and elution steps. | Automation-friendly, high reproducibility, high purity | Costly, requires specific equipment. Risk of contamination with residual beads | Magnetic rack, magnetic separator, magnetic beads. | High: Bead-based kits are often the most expensive | 30-50 | No |
| Liquid-Liquid Phase Separation | Thermal and pH-responsive capture and release of NAs in coacervates upon LLPS. | Simple separation with pH switch, standard equipment | Protocols and sample versatility are yet to be optimized. | Centrifuge, LLPS polymer, heat, buffers. | Inexpensive: LLPS polymer offers a high degree of scalability and the rest of reagents are standard. | 30-40 | No |

Table S2: Comparison of current NA extraction methods with potential optimized LLPS based method.

1. Chan, P. et al. Evaluation of extraction methods from paraffin wax embedded tissues for PCR amplification of human and viral DNA. *J Clin Pathol*. 54(5):401–403 (2001).
2. Boom, R. et al. Rapid and simple method for purification of nucleic acids. *J. Clin. Microbiol*. 28, 495–503 (1990).
3. Chomczynski, P. & Sacchi, N. Single-step method of RNA isolation by acid guanidinium thiocyanate–phenol–chloroform extraction*. Anal. Biochem*. 162, 156–159 (1987).
4. Kumar, A. & Garg, N. *Genetic engineering*. 101-102 (Nova Science Publishers, 2006).
5. Matson, R. S. *Microarray methods and protocols*. 27-29 (CRC Press, 2017).
6. He, H., Li, R., Chen, Y. et al. Integrated DNA and RNA extraction using magnetic beads from viral pathogens causing acute respiratory infections. *Sci Rep* 7, 45199 [10.1038/srep45199](https://www.nature.com/articles/srep45199)  (2017).
7. Possebon, F. S. et al. A fast and cheap in-house magnetic bead RNA extraction method for COVID-19 diagnosis. *J. Virol. Methods.* 300, 114414 [10.1016/j.jviromet.2021.114414](https://www.sciencedirect.com/science/article/pii/S0166093421003530?via%3Dihub) (2022).


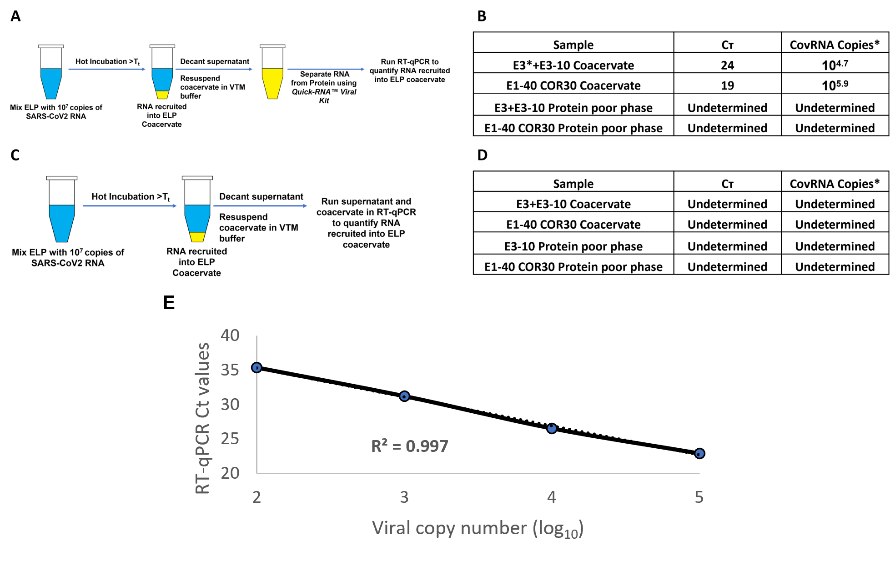


Figure S1: Quantification of SARS-CoV-2 RNA in complex coacervates and influence of ELP fusions on qPCR. **(A)** Workflow of the experiment designed to quantify the number of viral RNA copies that phase separate within the ELP coacervates. After LLPS, coacervates are resuspended in viral transfer media (VTM) and RNA is separated from protein using a commercial chromatographic method prior to RT-qPCR. **(B)** Estimation of viral RNA copies that were recruited into the ELP coacervates. C_T_ refers to cycle threshold and it is defined as the number of cycles necessary for the PCR to detect a specific genetic material. **(C)** Workflow of the experiment designed to recruit viral RNA into ELP coacervates for subsequent use in RT-qPCR. **(D)** RT-qPCR results of ELP-RNA rich solutions. **(E)** Standard curve prepared with 10-fold serial dilutions of viral SARS-CoV-2 RNA and obtained by regression analysis plotting the threshold cycle values (Ct) vs. the logarithm of the actual starting RNA copy number.

**
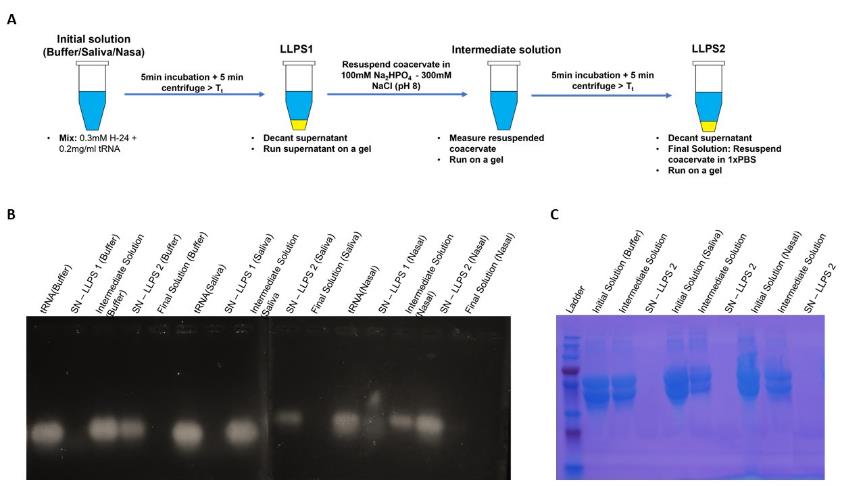
**

Figure S2: Recruitment of tRNA into protein coacervates upon H-24 LLPS in different solutions. **(A)** Workflow of two-step tRNA isolation assay. **(B)** 2.5% agarose gels stained with SyBr Gold illustrate the recruitment of tRNA into 0.5mM H-24 protein-rich phase at pH 6.5, and it subsequent release into the supernatant after LLPS2 at pH 8. **(C)** SDS-PAGE gel of two-step isolation assay to assess protein contamination.

**
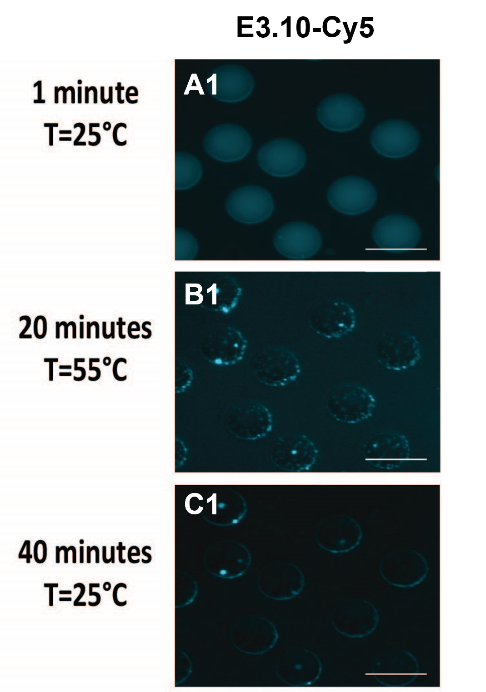
**

Figure S3: 10μM Cy5-E3.10 LLPS. Representative images of microfluidic generated droplets in fluorescence microscopy of **(A1)** soluble E3.10 below T_t_, **(B1)** phase separation of E3.10 into partially coarsened condensates above T_t_, and **(C1)** E3.10 cooled below T_t_.

**
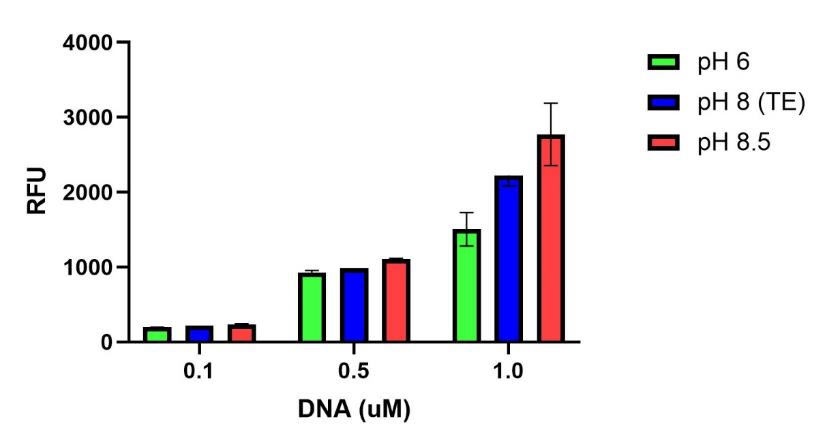
**

**Figure S4: Influence of pH in ATTO488-ssDNA1 intensity.** Measurement of 0.1, 0.5 and 1µM of fluorescently labeled ATTO488-ssDNA1 at pH 6, 8 and 8.5 to study the difference in fluorescence emission due to pH.

**
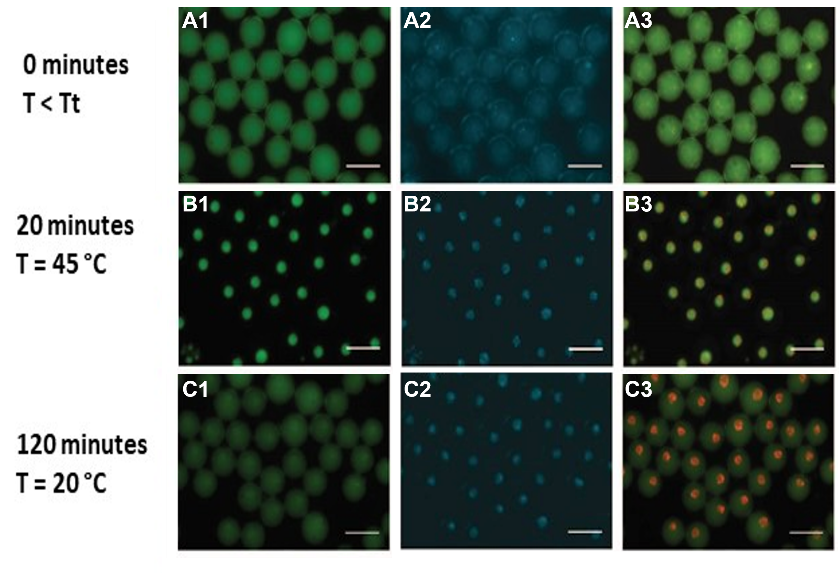
**

Figure S5: Photomicrographs for 0.5mM Alexa488-E3 + 10μM Cy5-E3.10 LLPS. Representative images of microfluidic generated droplets in fluorescence microscopy of **(A1-C1)** Alexa488 labeled E3 **(A2-C2)** Cy5 labeled E3.10 and **(A3-C3)** Merge of Alexa488 and Cy5 channel. Images show **(A1-A3)** soluble E3 and E3.10 below T_t_, **(B1-B3)** phase separation of E3.10 and E3 into fully coarsened condensates above T_t_, and **(C1-C3)** dissolution of E3 and E3.10 upon cooling below T_t_.

**
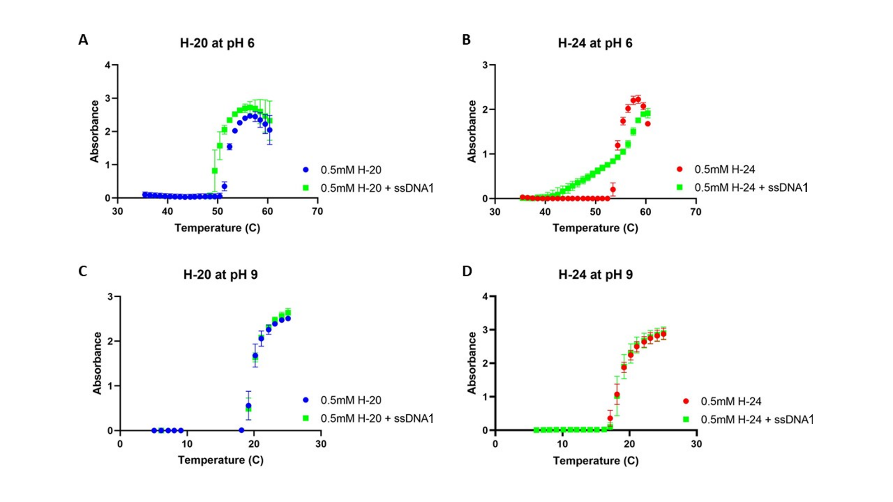
**

Figure S6: Temperature-dependent absorbance (turbidity) measurements for His-ELPs in aqueous buffers without and with ssDNA1 (raw data). Temperature-dependent absorbance measurements at 380nm from replicate measurements of **(A)** 0.5mM H-20 at pH 6, **(B)** 0.5mM H-24 at pH 6, **(C)** 0.5mM H-20 at pH 9 and **(D)** 0.5mM H-24 at pH 9, each without ssDNA1 and with ssDNA1.

**Uncropped gel images**


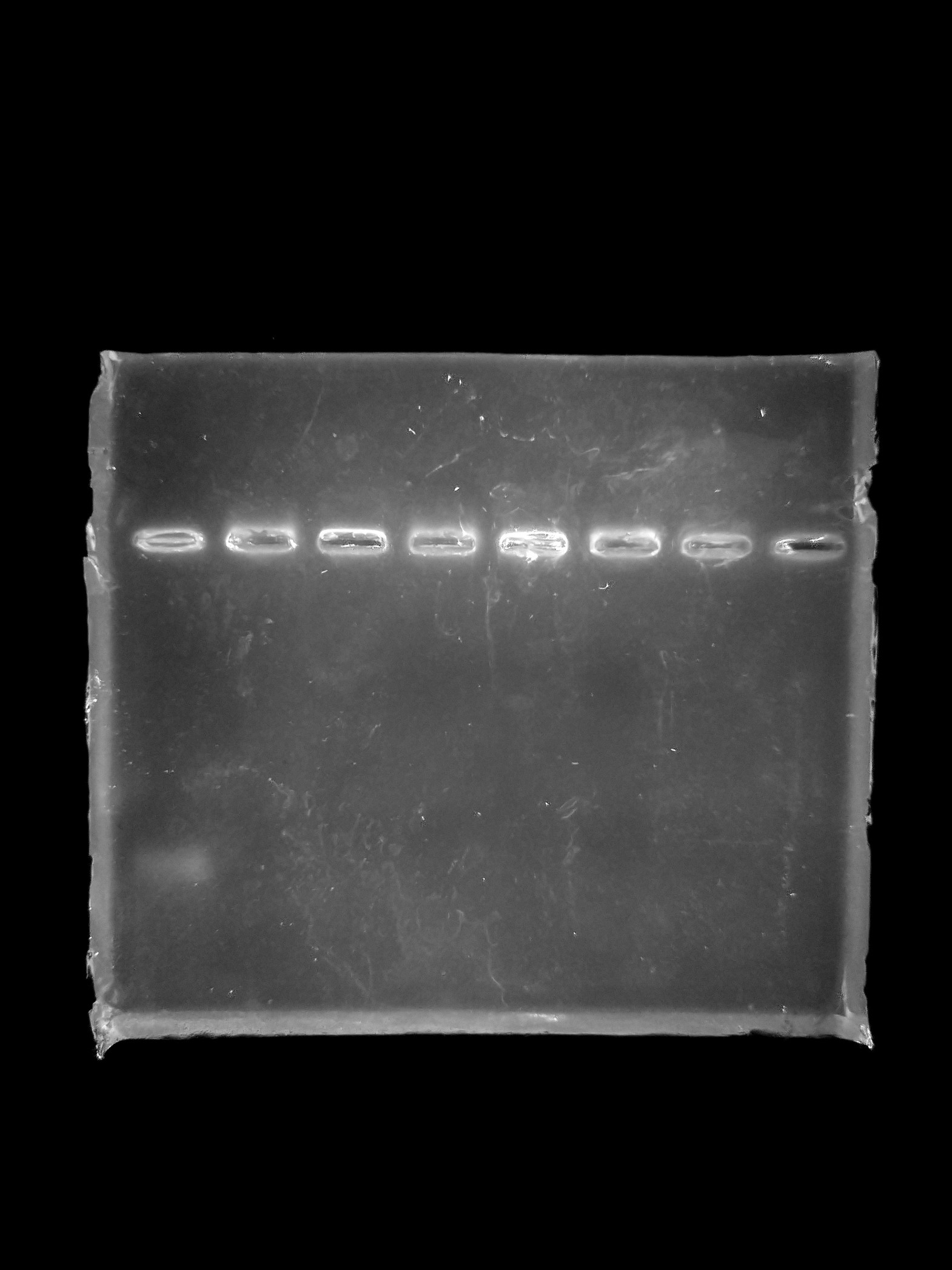


Figure S7: Uncropped image of gel for Figure 2A.


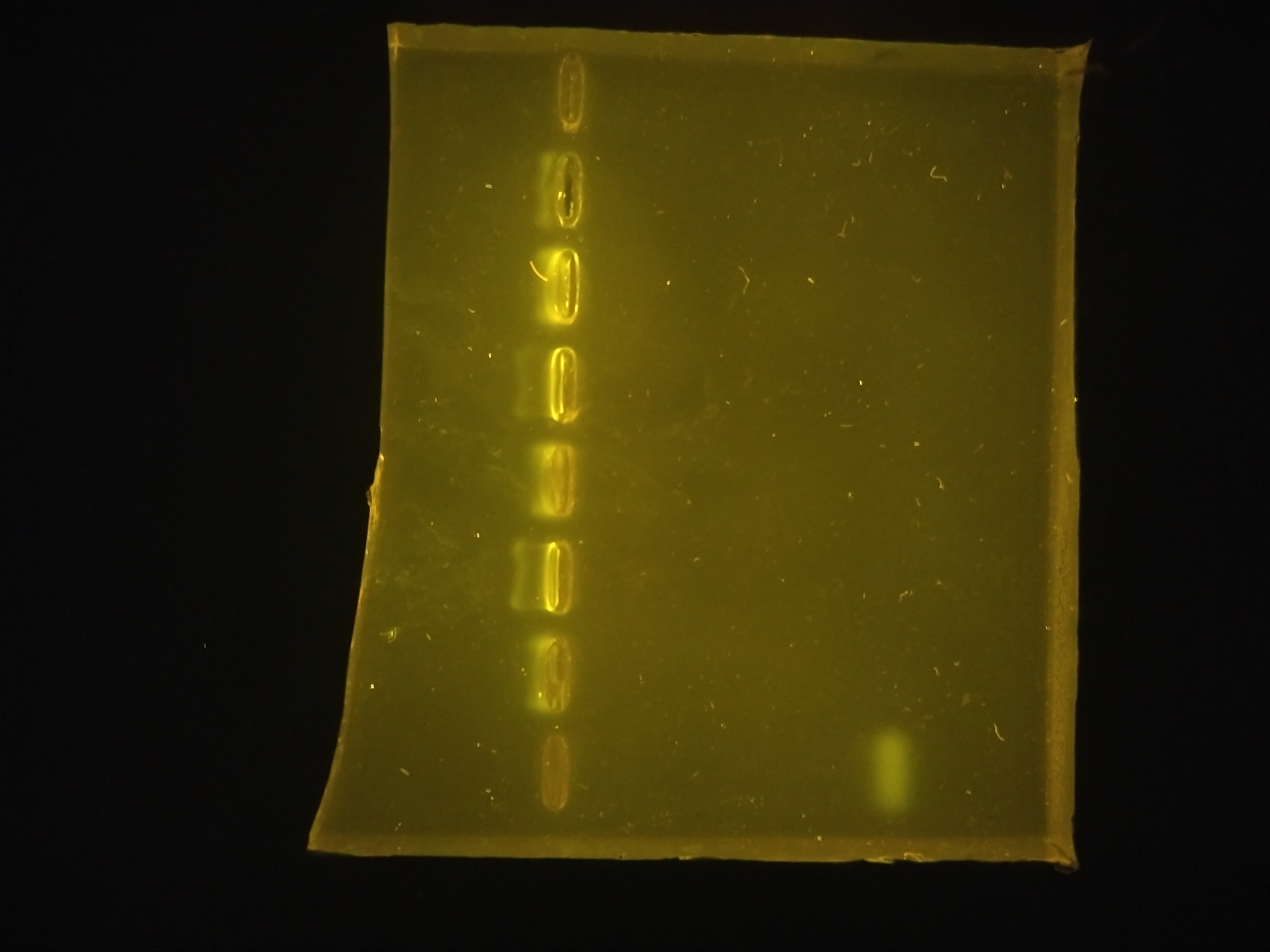


Figure S8: Uncropped image of gel for Figure 2B.


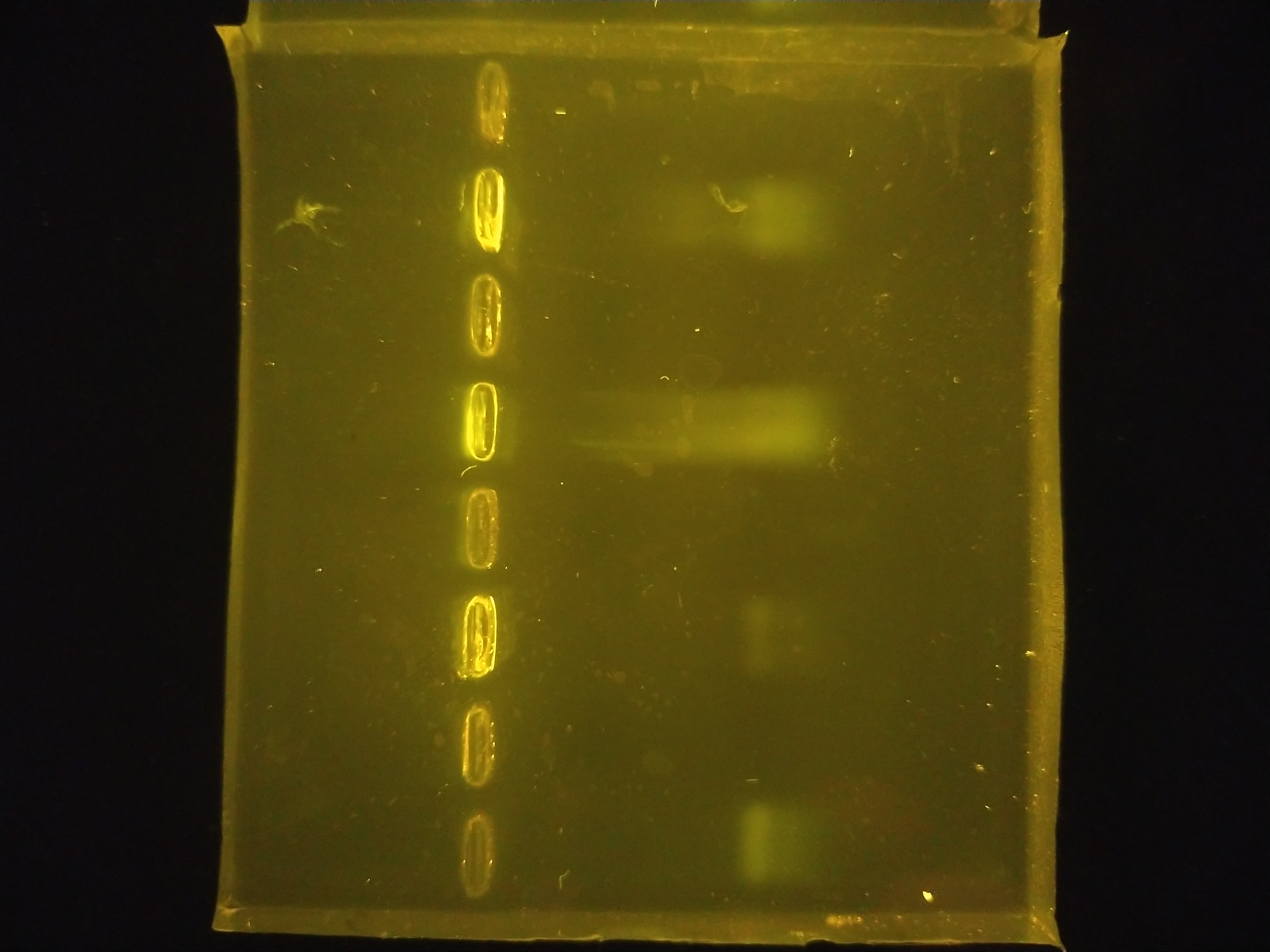


Figure S9: Uncropped image of gel for the first half of Figure 3B.


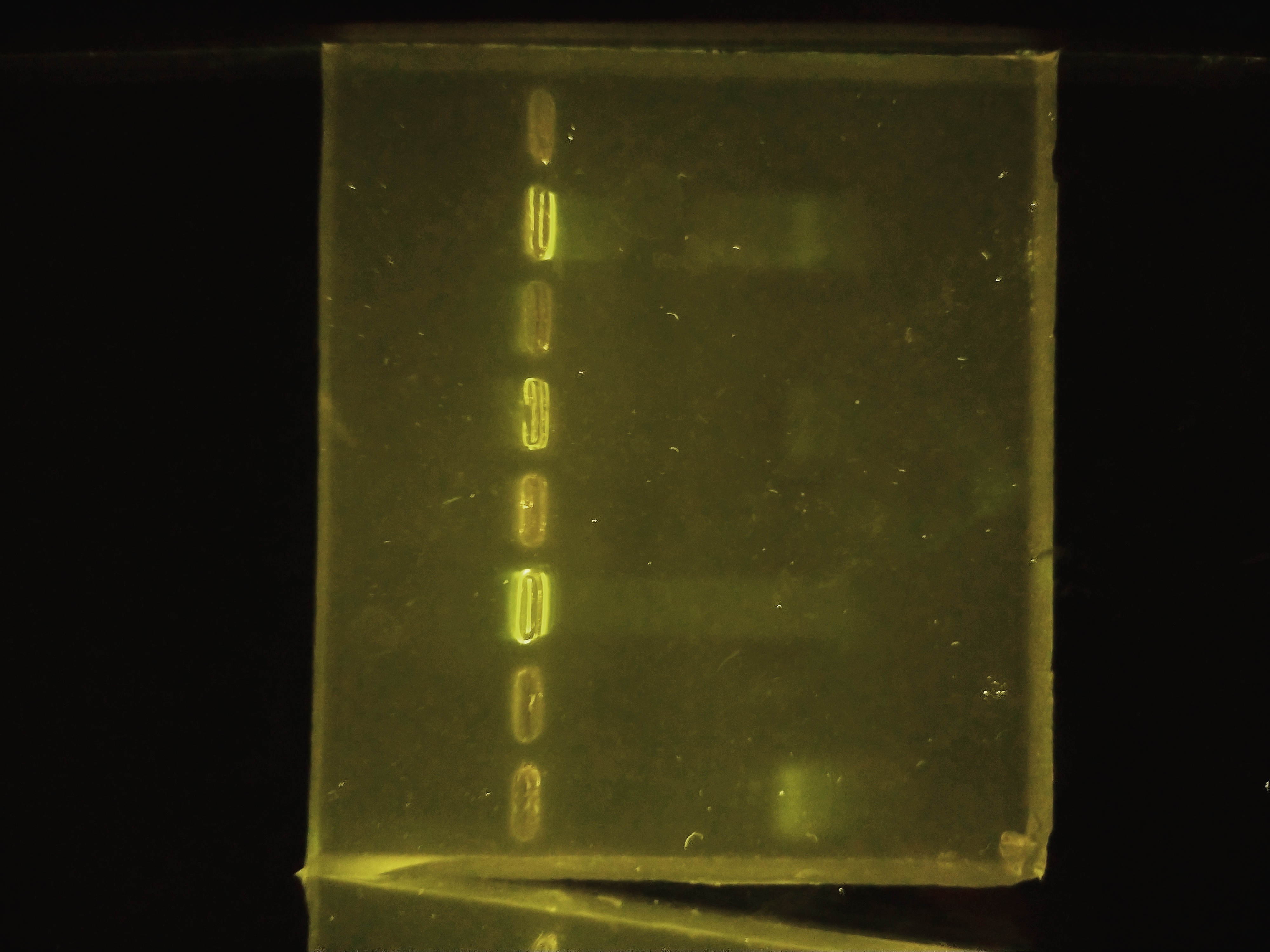


Figure S10: Uncropped image of gel for the second half of Figure 3B.


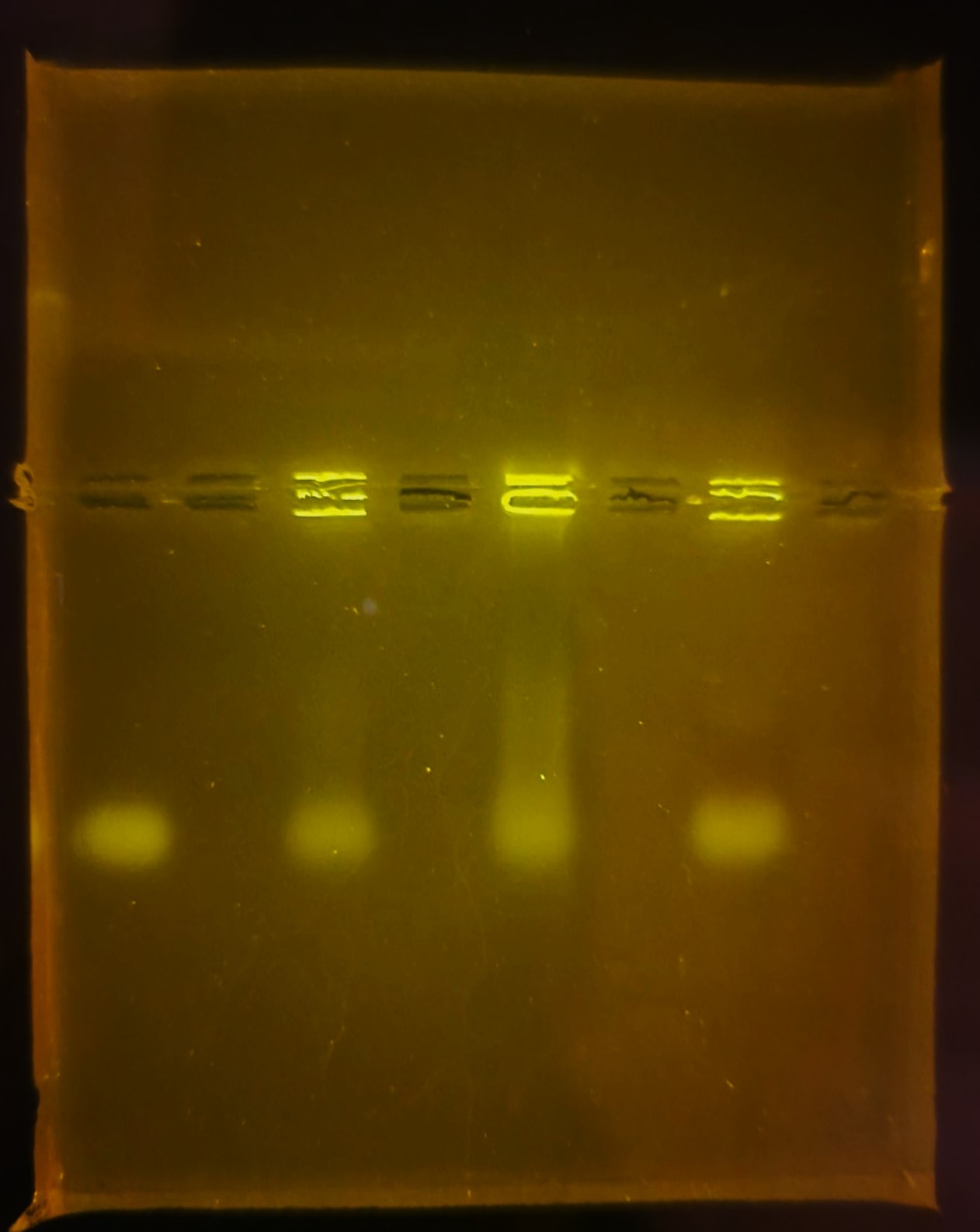


Figure S11: Uncropped image of gel for the first half of Figure 3C.


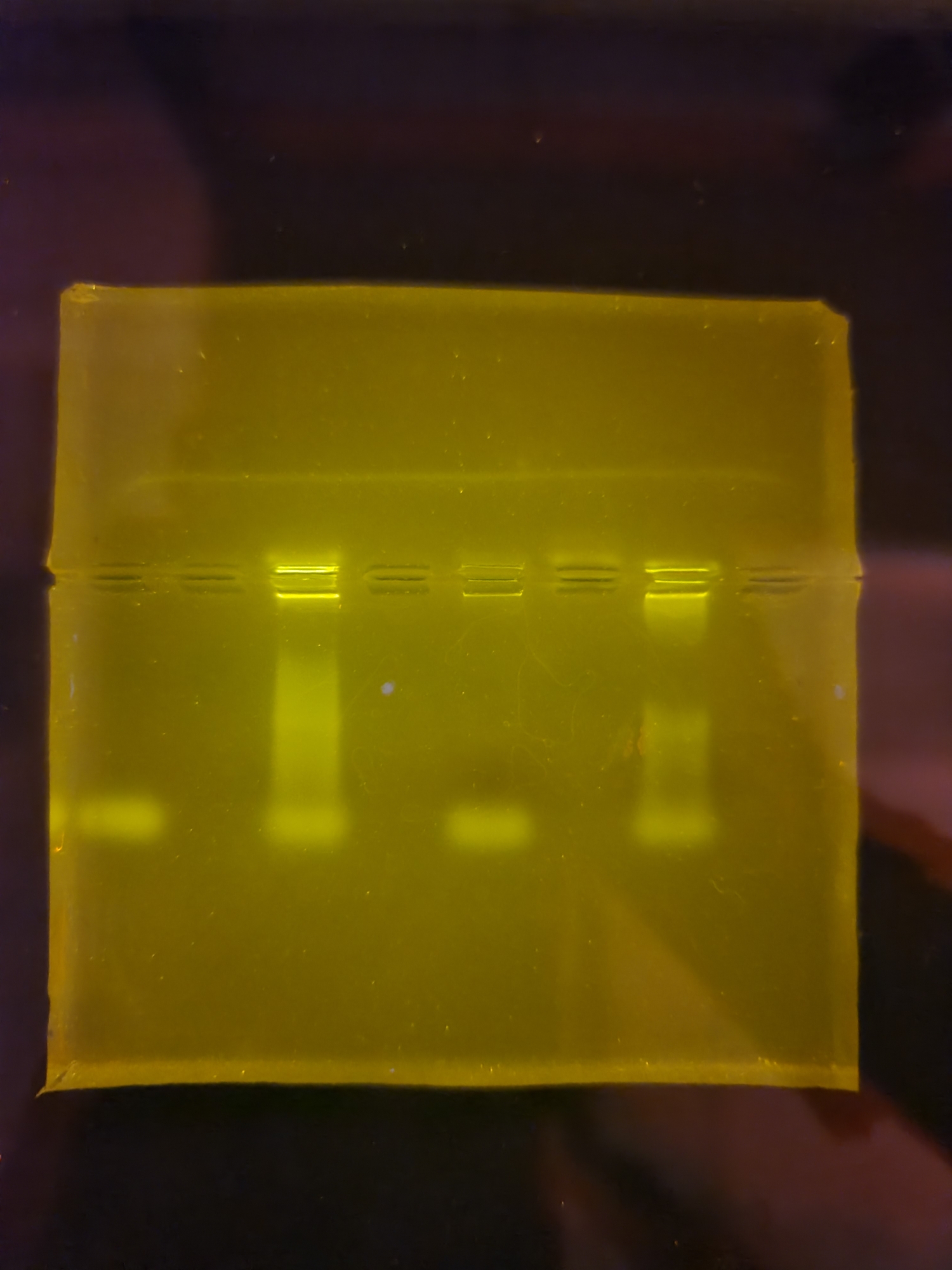


Figure S12: Uncropped image of gel for the second half of Figure 3C.


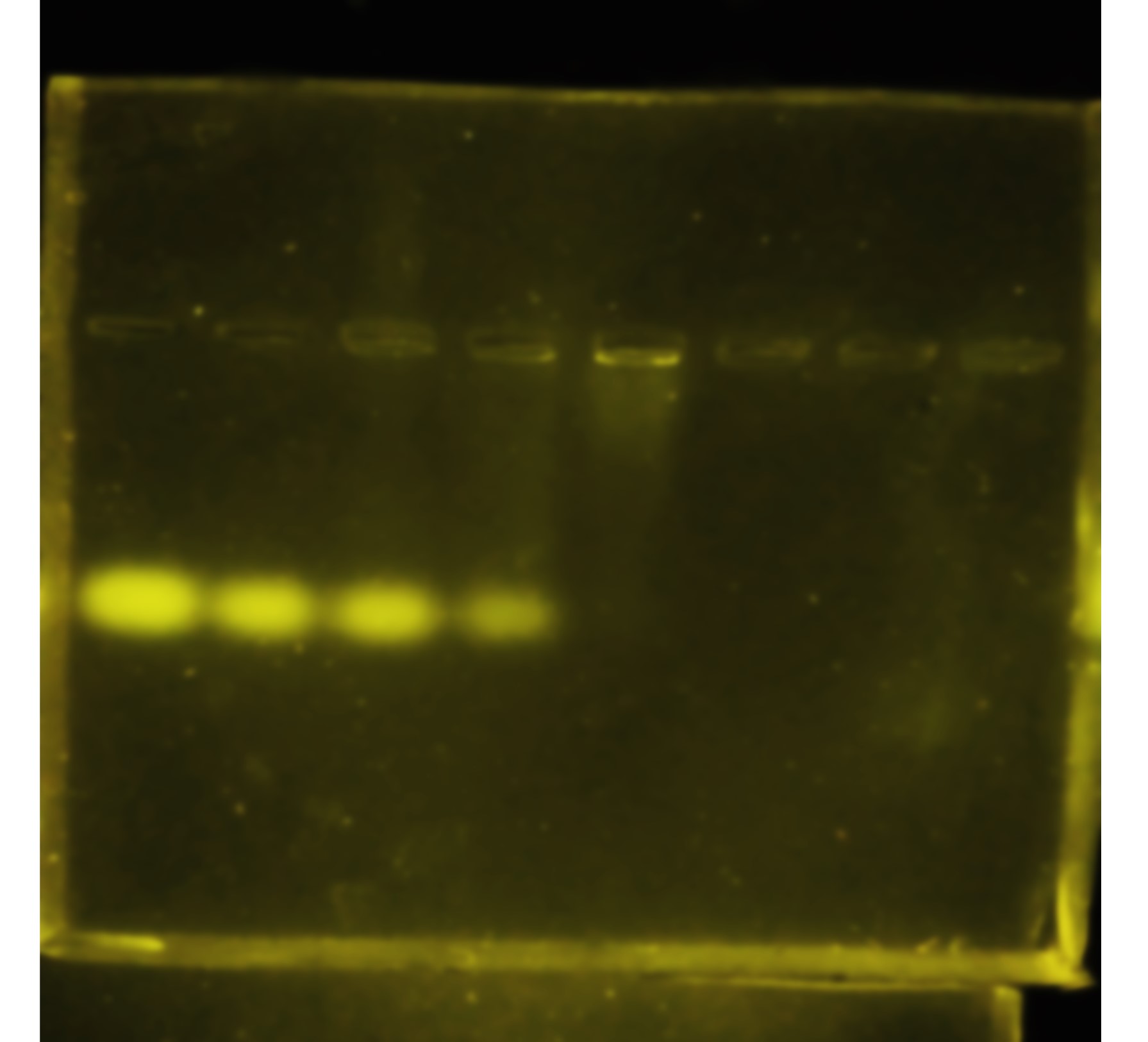


Figure S13: Uncropped image of gel for Figure 5 A1.


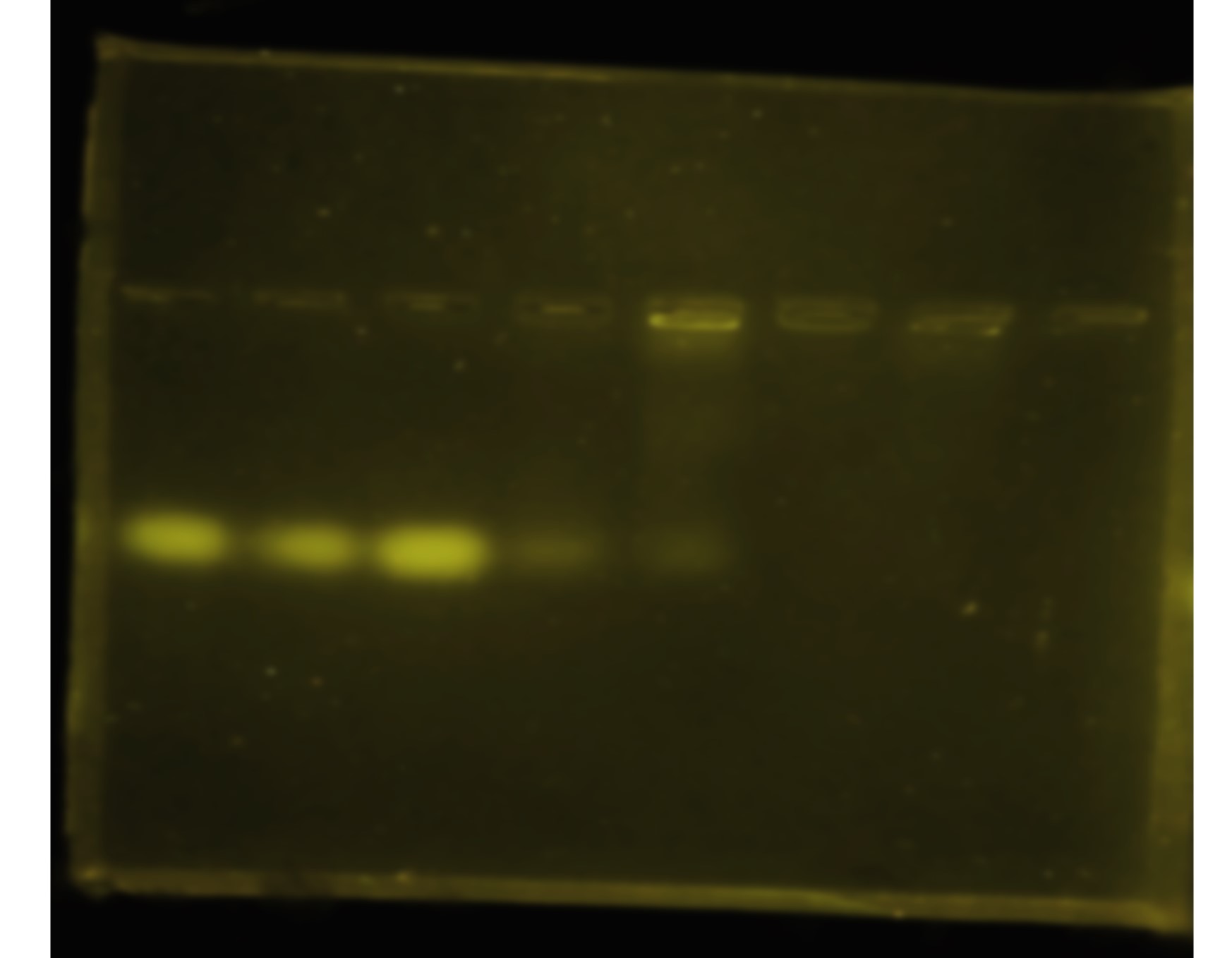


Figure S14: Uncropped image of gel for Figure 5 A2.


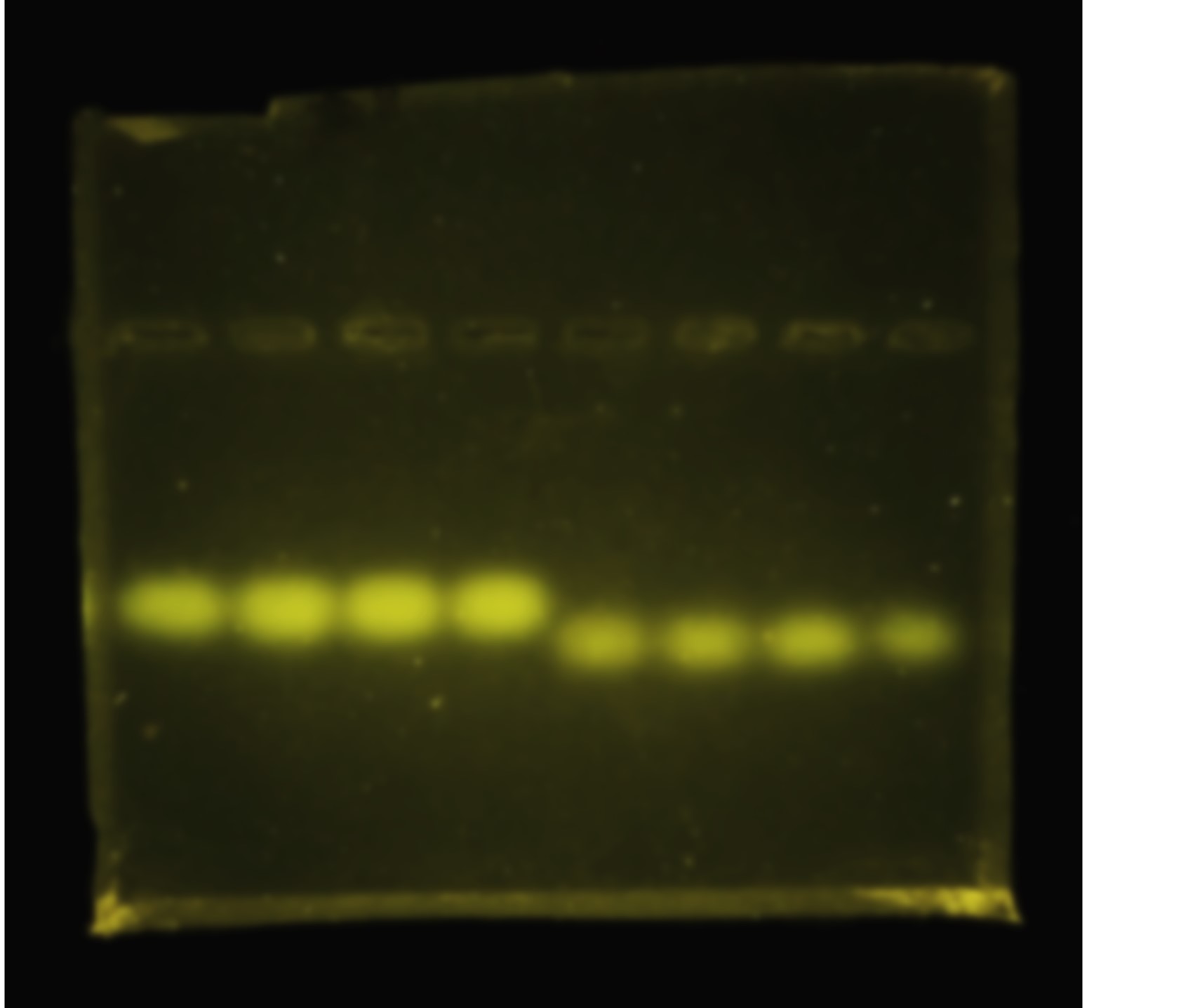


Figure S15: Uncropped image of gel for Figure 5 A3 (lanes 5-8 above).


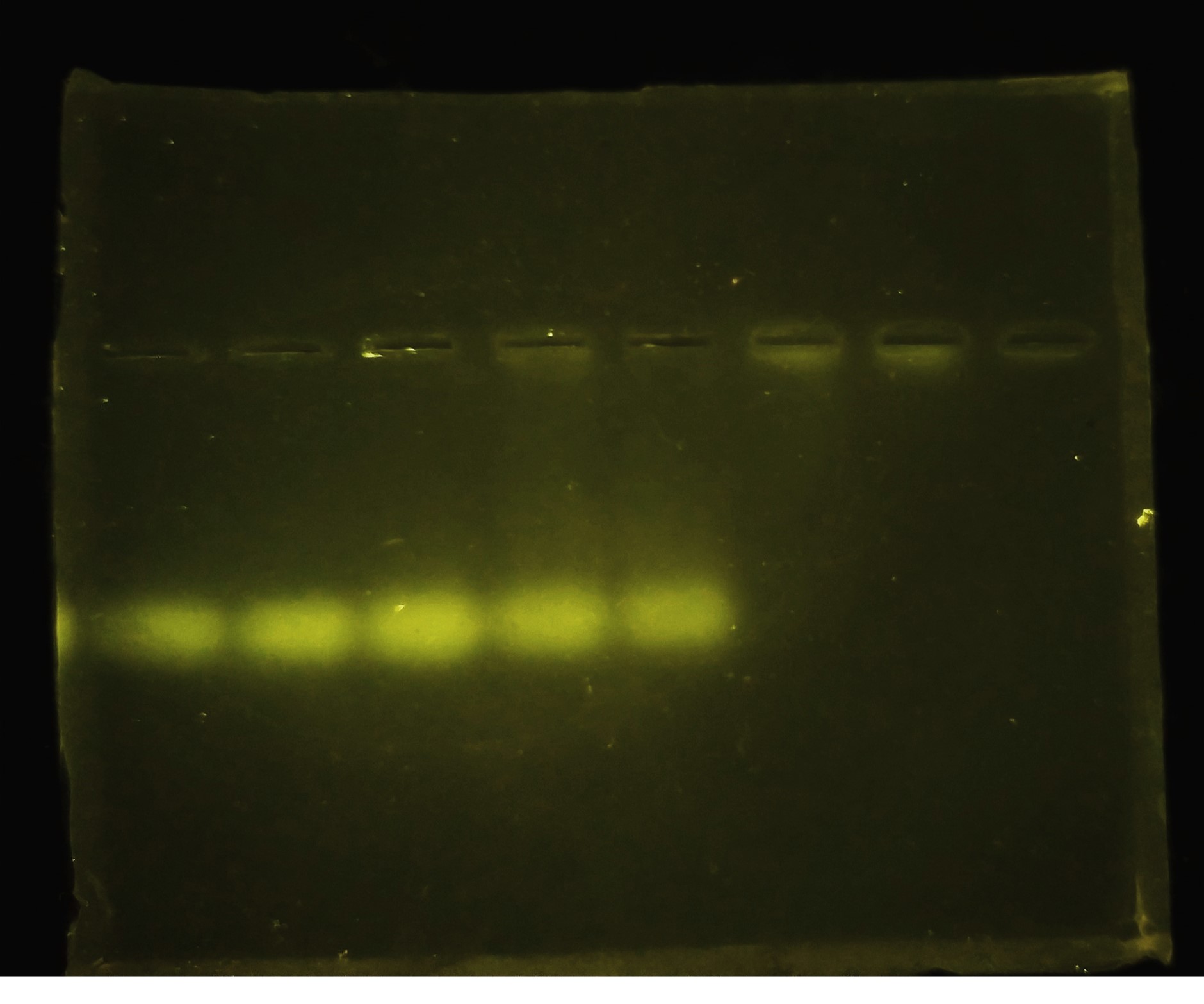


Figure S16: Uncropped image of gel for Figure 5 B1.


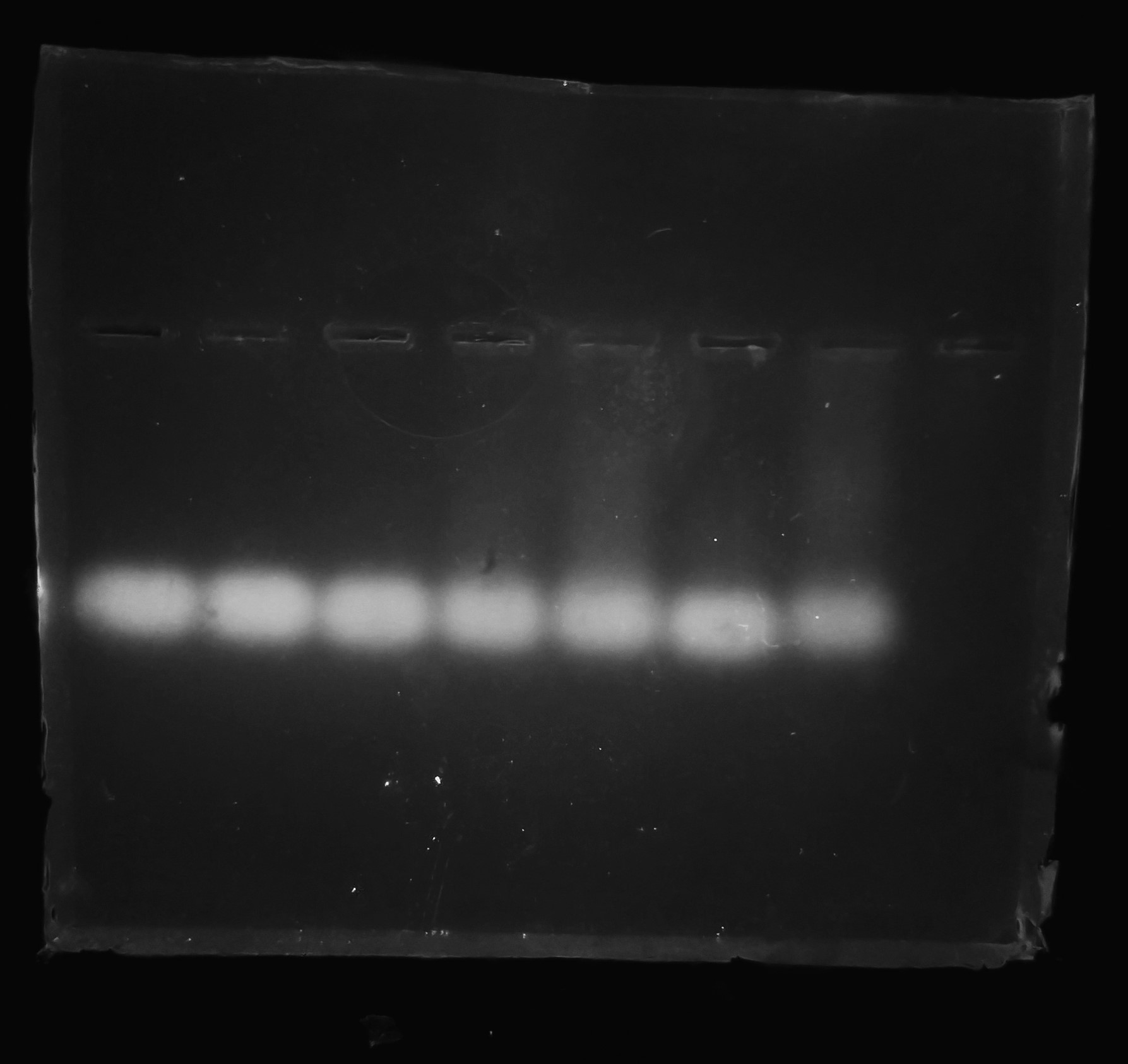


Figure S17: Uncropped image of gel for Figure 5 B2.


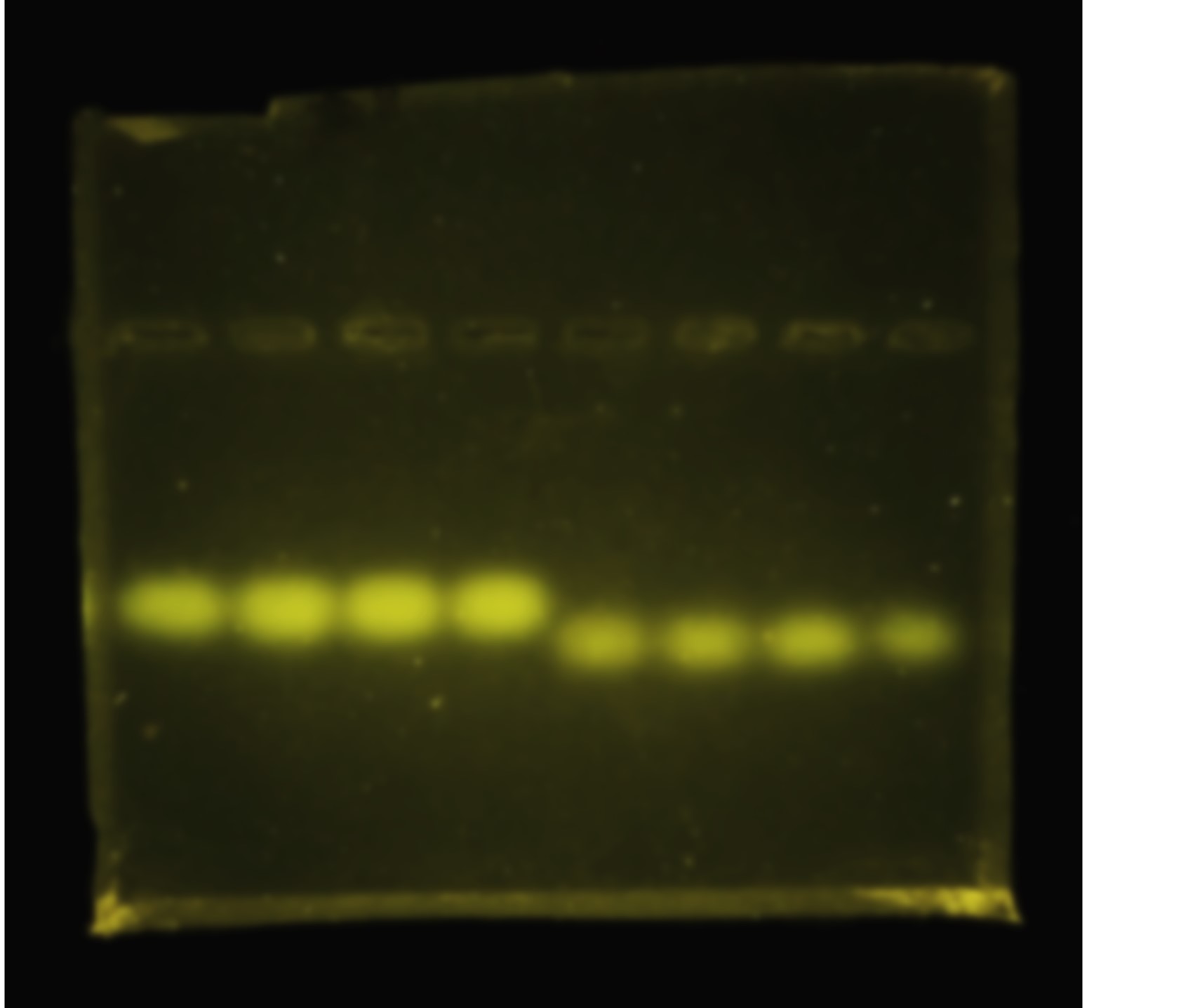


Figure S18: Uncropped image of gel for Figure 5 B3 (lanes 1-4 above).


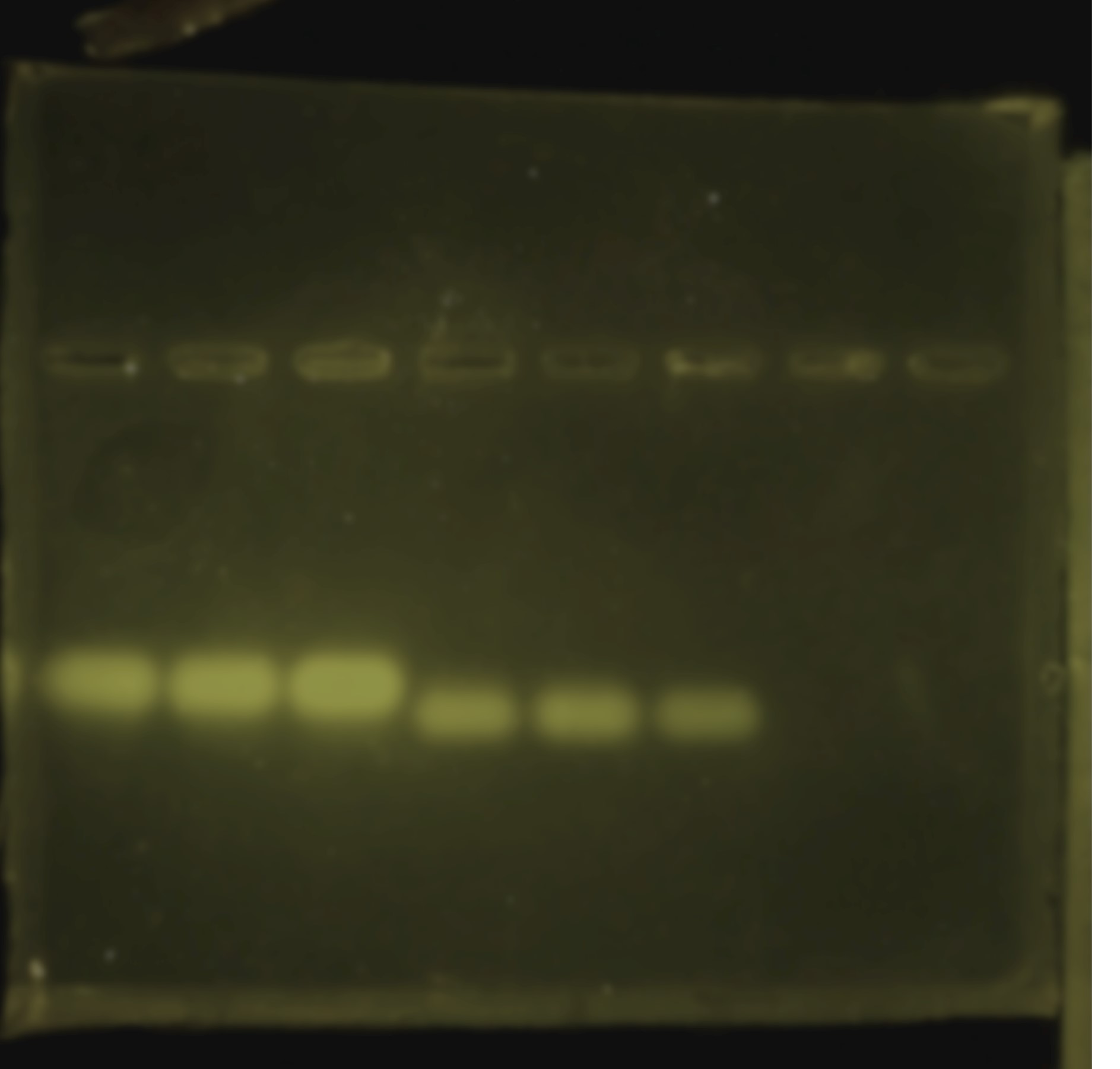


Figure S19: Uncropped image of gel for Figure 7.


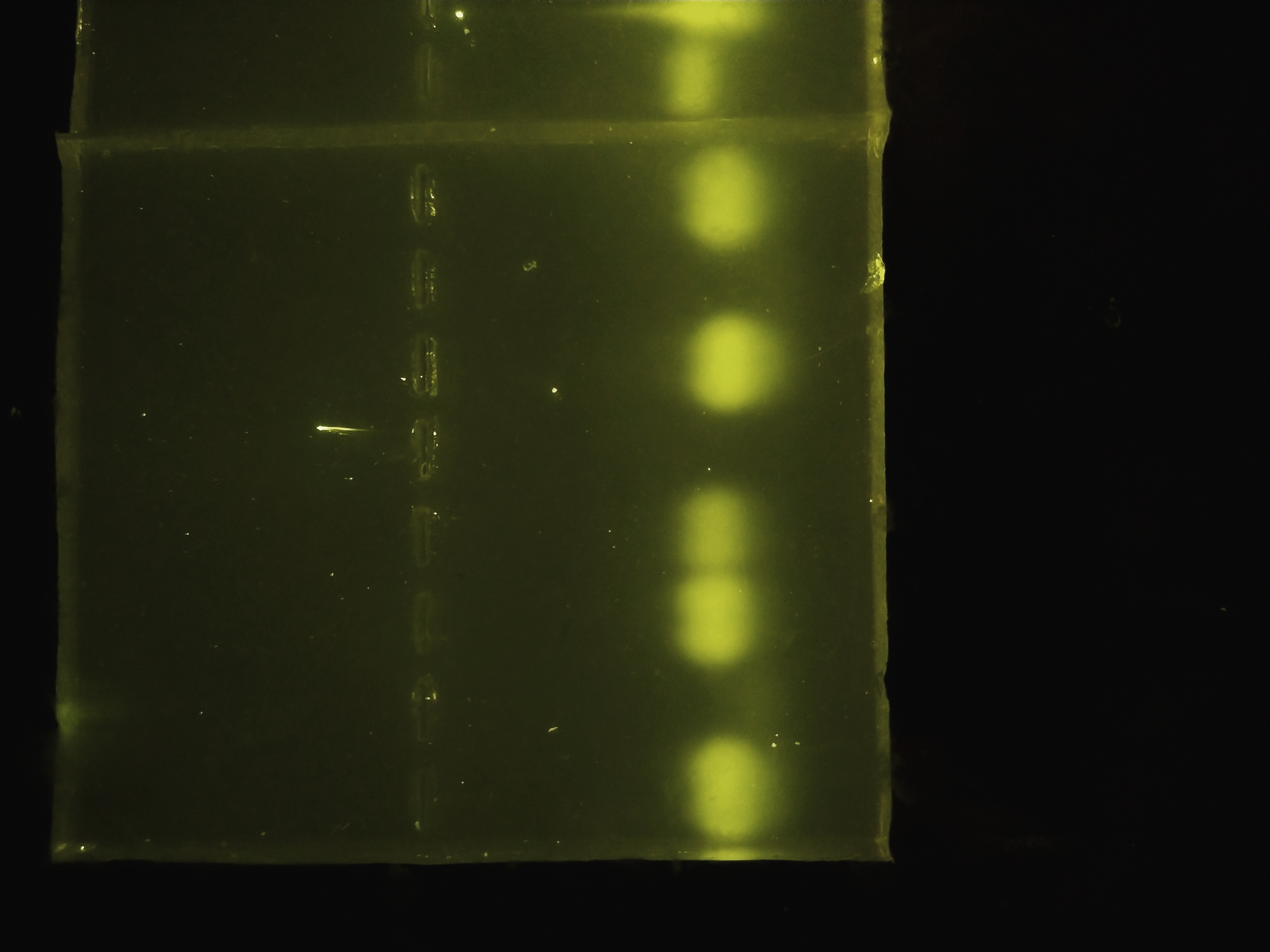


Figure S20: Uncropped image of gel for Figure S2B (left).


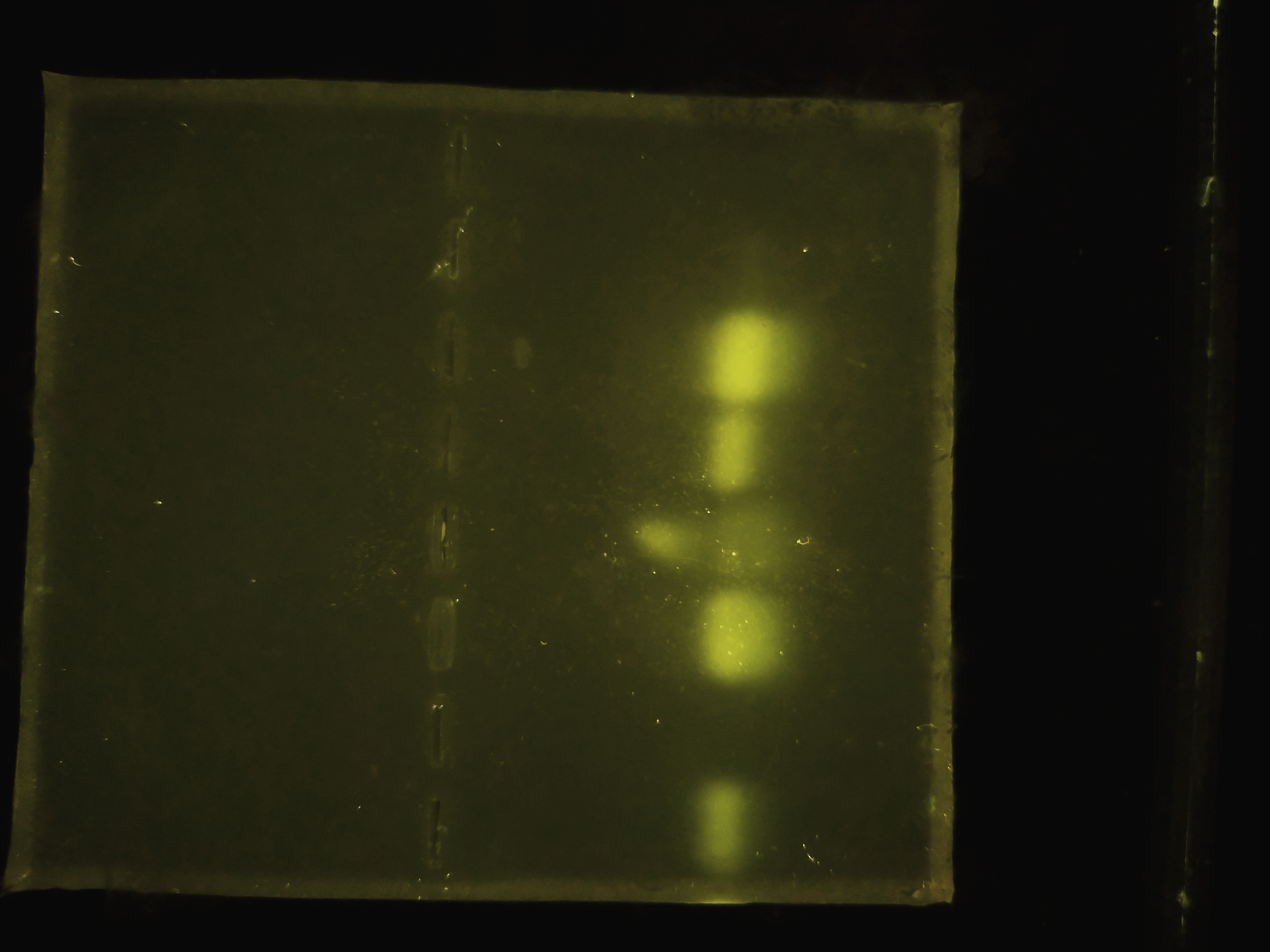


Figure S21: Uncropped image of gel for Figure S2B (right).


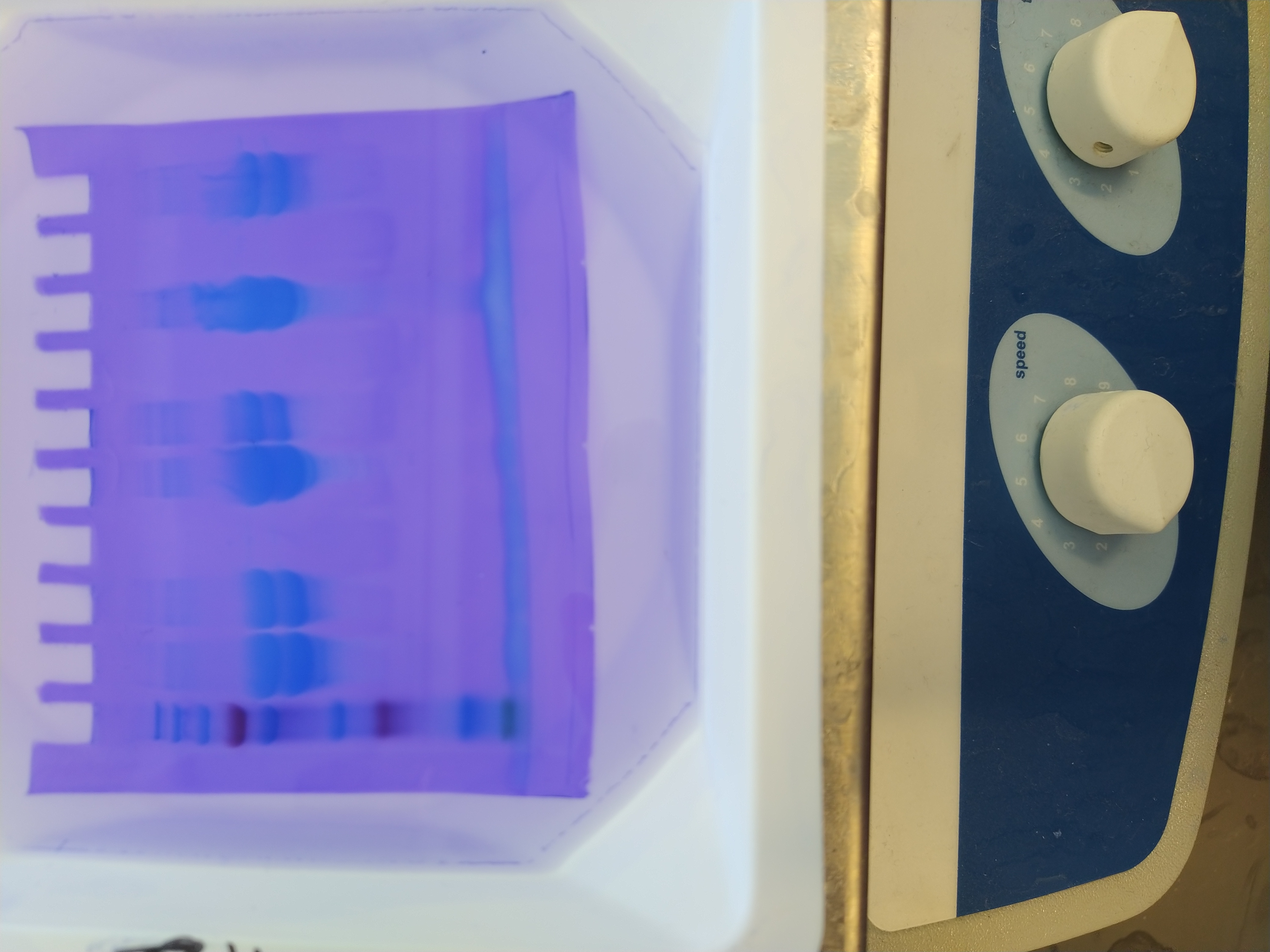


Figure S22: Uncropped image of gel for Figure S2C.
